# Rv1460, a SufR homologue, is a repressor of the *suf *operon in *Mycobacterium tuberculosis*

**DOI:** 10.1371/journal.pone.0200145

**Published:** 2018-07-06

**Authors:** Danicke Willemse, Brandon Weber, Laura Masino, Robin M. Warren, Salvatore Adinolfi, Annalisa Pastore, Monique J. Williams

**Affiliations:** 1 DST-NRF Centre of Excellence for Biomedical Tuberculosis Research; South African Medical Research Council Centre for Tuberculosis Research; Division of Molecular Biology and Human Genetics, Faculty of Medicine and Health Sciences, Stellenbosch University, Tygerberg, South Africa; 2 Electron Microscope Unit, University of Cape Town, Cape Town, South Africa; 3 Structural Biology Science Technology Platform, The Francis Crick Institute, London, United Kingdom; 4 Pharmaceutical Science and Technology, University of Turin, Turin, Italy; 5 Department of Basic and Clinical Neuroscience, Maurice Wohl Institute, King's College London, London, United Kingdom; National Institute of Child Health and Human Development, UNITED STATES

## Abstract

Iron–sulphur (Fe-S) clusters are ubiquitous co-factors which require multi-protein systems for their synthesis. In *Mycobacterium tuberculosis*, the *Rv1460-Rv1461-Rv1462-Rv1463-csd-Rv1465-Rv1466* operon (*suf* operon) encodes the primary Fe-S cluster biogenesis system. The first gene in this operon, *Rv1460*, shares homology with the cyanobacterial SufR, which functions as a transcriptional repressor of the *sufBCDS* operon. Rv1460’s function in *M*. *tuberculosis* has however not been determined. In this study, we demonstrate that *M*. *tuberculosis* mutants lacking a functional Rv1460 protein are impaired for growth under standard culture conditions. Elevated expression of *Rv1460* and *Rv1461* was observed in the mutant, implicating Rv1460 in the regulation of the *suf* operon. Binding of an Fe-S cluster to purified recombinant Rv1460 was confirmed by UV-visible spectroscopy and circular dichroism. Furthermore, three conserved cysteine residues, C203, C216 and C244, proposed to provide ligands for the coordination of an Fe-S cluster, were shown to be required for the function of Rv1460 in *M*. *tuberculosis*. Rv1460 therefore seems to be functionally analogous to cyanobacterial SufR.

## Introduction

*Mycobacterium tuberculosis*, the causative agent of tuberculosis (TB), accounts for nearly 1.5 million deaths globally each year and therefore remains an important pathogen [1]. Following inhalation, *M*. *tuberculosis* is phagocytosed by alveolar macrophages where it encounters numerous stresses such as nutrient limitation, low pH, reactive oxygen (ROS) and reactive nitrogen species (RNS) [2]. Subsequent immunological containment of infected macrophages within granulomas creates a hypoxic environment [3,4], which triggers a metabolic downshift in the bacteria to a non-replicating state [5,6]. Bacteria in this non-replicating state are refractory to killing by antibiotics [5], necessitating extended chemotherapy with multiple drugs in order to achieve sterilisation in patients infected with *M*. *tuberculosis*.

In addition to hypoxia, iron starvation was recently shown to induce a metabolic downshift in *M*. *tuberculosis* in vitro, and was proposed as an important trigger for entering a non-replicating state during infection [7]. While this metabolic downshift was associated with a general repression of gene expression, induction of a small set of genes was also observed. These genes included those involved in iron-sulphur (Fe-S) cluster biogenesis and critical Fe-S cluster containing enzymes. Fe-S clusters are ubiquitous co-factors required by enzymes involved in several critical cellular processes, and for regulating gene expression by modulating the binding of certain transcriptional regulators to DNA [8]. In vivo, multi-protein systems are required for the assembly of Fe-S clusters, and three highly conserved systems, namely the nitrogen fixation (NIF) [9], Fe-S cluster (ISC) [10] and the sulphur mobilisation (SUF) systems [11] have been identified. In *Escherichia coli* the ISC system functions as the house-keeping system [12], while the SUF system is induced under conditions of oxidative stress and iron limitation [13–15]. The genomes of most gram-positive bacteria encode only a SUF system, and in some cases, unique configurations of these systems are present. The *M*. *tuberculosis* genome encodes a single gene cluster with homology to the SUF system, namely *Rv1460-Rv1461-Rv1462-Rv1463-csd-Rv1465-Rv1466*, which is transcribed as an operon (*suf* operon) [16]. The *suf* operon encodes the primary Fe-S cluster assembly system in mycobacteria [17] and all the genes in the operon, with the exception of *Rv1460*, are predicted to be essential for in vitro growth of *M*. *tuberculosis* [18,19].

The first step in Fe-S cluster assembly involves the mobilisation of sulphur from cysteine by a cysteine desulphurase. The genome of *M*. *tuberculosis* encodes three genes with homology to cysteine desulphurases, namely *csd*, located in the *suf* operon, *iscS* and *Rv3700c* [20]. IscS_MTB_ is proposed to play a role in the assembly of Fe-S clusters directly on apo-proteins, since no other ISC system components are present in mycobacteria [16,21]. *Rv3700c* encodes a cysteine-serine lyase (EgtE) involved in the synthesis of the low molecular weight thiol, ergothioneine [22]. Following sulphur mobilisation, the Fe-S cluster is assembled on a scaffold. In the SUF system, the SufBCD complex functions as a scaffold for *de novo* Fe-S cluster assembly [23–26] and in *M*. *tuberculosis*, *Rv1461*, *Rv1462* and *Rv1463* encode the SufB, SufC and SufD homologues respectively [16]. A role for SufD in iron acquisition [27] and SufC in providing energy for conformational changes in the SufB/D interface during cluster assembly has been proposed [13,23]. These functions are however yet to be confirmed for the mycobacterial proteins. Rv1461 contains an intein [28–30], which must be spliced before interaction with Rv1462 and Rv1463 can occur [28]. Intein splicing is sensitive to oxidative and nitrosative stress and may function to regulate cluster assembly in response to the environment [30].

The expression of Fe-S cluster biogenesis systems requires tight regulation since excessive Fe-S clusters are toxic and inhibit bacterial growth [11], while inadequate expression has deleterious effects on Fe-S cluster containing enzymes and regulators. Several studies have demonstrated induction of the *suf* operon in *M*. *tuberculosis* in response to stress conditions that are likely to increase the need for cluster repair or de novo synthesis, including exposure to hydrogen peroxide, diamide [2,31] and nitric oxide (NO) [31]. In addition, induction of *Rv1460*, *Rv1461*, *Rv1462*, *Rv1463* and *csd* was observed after 96 hours of nutrient starvation [32], while *Rv1461*, *Rv1462*, *csd*, *Rv1465* and *Rv1466* were induced upon infection of naïve and activated macrophages [2]. Gene expression analysis in sputum from TB patients revealed a 28-fold induction of *Rv1460* relative to in vitro expression, supporting a role for this regulator during infection [33]. The molecular mechanism by which expression of the *suf* operon is regulated is however currently unclear.

The first gene in the *suf* operon, *Rv1460*, encodes a probable transcriptional regulator of the *suf* system which shares homology with the cyanobacterial SufR transcriptional repressor [34]. In Synechocystis, *sufR* is divergently transcribed from the *sufBCDS* operon, and acts as a transcriptional repressor of *sufBCDS* expression and an auto-regulator of its own expression [34,35]. Two distinct DNA-binding sites for SufR have been identified in the region between the *sufR* gene and the *sufBCDS* operon, namely a high affinity site upstream of *sufBCDS* and a low affinity site upstream of *sufR*. Each homodimer of SufR coordinates two oxygen sensitive Fe-S clusters, and the presence as well as the redox-state of these clusters alter the affinity with which SufR binds to DNA [35]. Sequence alignment of Rv1460 with Synechocystis SufR demonstrated conservation of the cysteine residues that provide ligands to coordinate the Fe-S cluster, suggesting that Rv1460 is also an Fe-S cluster containing transcriptional regulator [35].

In this study, we demonstrate that loss of Rv1460 is deleterious for *M*. *tuberculosis* in vitro and confirm its function as a repressor of *suf* operon expression. Furthermore, we show that Rv1460 binds an Fe-S cluster and interrogate the role of the three cysteine residues predicted to coordinate the Fe-S cluster in the function of this regulator.

## Materials and methods

### Bioinformatic analysis

The sequence of Rv1460 was obtained from the Tuberculist database (http://tuberculist.epfl.ch) and homologues were identified by BLASTP analysis (http://blast.ncbi.nlm.nih.gov/Blast.cgi). Protein alignments were performed using the Maestro Multiple Sequence Viewer multiple alignment tool.

### Bacterial strains and culture conditions

*M*. *tuberculosis* H37Rv and *Mycobacterium smegmatis* mc^2^155 were cultured in 7H9 broth (Difco) with 0.05% Tween-80 supplemented with 0.2% glycerol and ADC (Bovine Albumin fraction V (50 g/L), dextrose (20 g/L) and catalase (0.0375 g/L)) or Middlebrook OADC (oleic acid ADC), or on Middlebrook 7H10 (Difco) supplemented with ADC or OADC. Kanamycin (50 μg/ml), hygromycin (50 μg/ml), 5-Bromo-4 Chloro-3 Indolyl β-D-galactosidase (X-gal) (40 μg/ml) and sucrose (5%) were used for selection purposes when applicable. *E*. *coli* XL1 Blue strain was used for cloning, while recombinant protein expression was performed using the *E*. *coli* Arctic express (DE3) (Agilent Technologies) and the BL21(DE3)pLysS (Novagen) strains. Culturing of *E*. *coli* was performed using LB broth and agar containing ampicillin (100 μg/ml), kanamycin (50 μg/ml), hygromycin (150 μg/ml), gentamycin (20 μg/ml), chloramphenicol (34 μg/ml) and X-gal (40 μg/ml) when applicable. Zinc sulphate (8.3 μM) was added to media during protein production. The bacterial strains used during this study are indicated in Table A in S1 File.

For iron limitation experiments metal defined media was prepared containing 0.5% asparagine, 0.5% KH_2_PO_4_, 0.2% glycerol, 0.05% Tween-80 and 10% ADC. The pH was adjusted to 6.8, and metal contamination removed by treatment with 5% Chelex-100 (Bio-Rad) for 24 hours. The media was then supplemented with 0.5 mg/l ZnSO_4_, 0.1 mg/l MnSO_4_ and 40 mg/l MgSO_4_, with (MM + Fe^3+^) or without (MM) 50 μM FeCl_3_ [36,37]. MM was previously shown to contain approximately 2 μM Fe^3+^ [37]. *M*. *tuberculosis* was pre-cultured in 7H9 OADC to mid-log growth phase. Cells were harvested by centrifugation, washed in equal volume of MM and sub-cultured into MM or MM + Fe^3+^ to a starting OD_600nm_ of 0.05 and cultured to late log phase. Two subsequent growth cycles were performed in MM or MM + Fe^3+^.

### Transcriptional start site determination for *Rv1460* and *Rv1461*

The transcriptional start site of *Rv1460* and *Rv1461* were determined using the adaptor- and radioactivity-free identification of transcriptional start sites (ARF-TSS) method as previously described [38]. Briefly, 500 ng of RNA was converted to cDNA using phosphorylated primers, 1460ARF-TSS or 1461ARF-TSS (Table B in S1 File). Following circularisation of cDNA with T4 RNA ligase, PCR was performed using the 1460TSS-F1 and 1460TSS-R1 or 1461TSS-F1 and 1461TSS-R1 primer sets (Table B in S1 File). The resulting product was cloned into the pJET1.2 vector for Sanger sequencing.

### Generation of *E*. *coli* and mycobacterial expression vectors and β-galactosidase reporter plasmids

Vectors for the overexpression of Rv1460 as a N-terminally His-tagged protein were generated as indicated in Table C in S1 File. *Rv1460* was amplified using the primers indicated in Table D in S1 File and cloned into pSE100 (hygromycin) to generate pSE1460 (Table C in S1 File). The three cysteine residues, C203, C216 and C244, predicted to coordinate an Fe-S cluster in Rv1460 [35] and an additional cysteine residue, C242, were replaced with serine residues to create Rv1460_C203S, Rv1460_C216S, Rv1460_C242S and Rv1460_C244S protein variants. Site directed mutagenesis was performed using a previously described protocol [39] with pJET1.2Rv1460N as a template, and the mutagenic primers indicated in Table D in S1 File. Three rounds of site directed mutagenesis was performed to generate the Rv1460_C203S/C216S/C244S variant. The mutated fragments were then sub-cloned into pET28a. Each pET28 vector harbouring mutations in Rv1460 (Table C in S1 File) was digested with SfiI and HindIII and the resulting 486 bp gene fragment cloned into pSE1460 digested with the same enzymes, to generate pSE1460_C203S, pSE1460_C216S, pSE1460_C242S, pSE1460_C244S and pSE1460_C203S/C216S/C244S. A series of mycobacterial expression vectors conferring kanamycin resistance were generated by sub-cloning the promoter and gene (BcuI/SalI digested) from each pSE1460 vector into the pCV-125 backbone (EcoRV/ BstZ171 digested). β-galactosidase reporter plasmids were generated by cloning either a 139 bp or a 314 bp fragment into pJEM15 as described in Tables C and D in S1 File.

### β-galactosidase promoter activity assays

*M*. *smegmatis* culture (5 ml) (OD_600nm_ between 0.4 and 0.6), was washed with phosphate buffered saline (PBS) containing 0.05% Tween-80, the cells pelleted by centrifugation and stored at -80°C until the assay was performed. Cells were re-suspended in 2 ml of 10 mM Tris-HCl (pH 8.0) and ribolysed for four 25 s cycles at 4.5 watt. The extract was then clarified by centrifugation at 21 130 × g for 10 min at 4°C. *ortho*-Nitrophenyl-β-galactoside (ONPG) was dissolved in Z-buffer (60 mM Na_2_HPO_4_, 40 mM NaH_2_PO_4_, 10 mM KCl, 1 mM MgSO_4_) to a final concentration of 1.33 mg/ml and mixed with an equal volume of whole cell lysate to initiate the assay. The absorbance at 420 nm was monitored over a period of 30 min and Miller units calculated as follows: 200 × (change in OD_450nm_) per mg protein per min. The amount of protein per sample was calculated using the BioRad protein assay.

### Generation of *M*. *tuberculosis* H37Rv strains harbouring unmarked deletions of the *Rv1460* gene

Two-step allelic exchange [40] was used to generate two different Rv1460 deletions; Δ*Rv1460*, which has an in frame deletion of the bulk of the Rv1460 gene (codons 36 to 211 deleted), and Rv1460 truncation mutant (*Rv1460*stop) which has a DNA-binding domain deletion and a premature stop codon at position 122. Details of the primers and plasmids used for the generation of allelic exchange vectors and further description of the mutants are indicated in Tables C and D in S1 File. PCR and Southern blotting was used to confirm the presence of the deletions as described in Table E and Figures B-D in S1 File. The three truncation mutants (Δ*Rv1460*stop_1.19, Δ*Rv1460*stop_5.19 and Δ*Rv1460*stop_5.20) were generated using the same allelic exchange substrate. PCR and Sanger sequencing was used to confirm that the deletion introduced into the three strains was identical. Sanger sequencing also confirmed that the deletion introduced would result in a frame-shift and truncation of Rv1460 in all three mutants (Figure D in S1 File). The mutants were complemented using an integrating vector or a stable integrating vector, in which the integrase gene was removed, containing the *Rv1460* gene and 139 bp upstream promoter region [41], as indicated in Table C in S1 File.

### RT-qPCR

Mid-log *M*. *tuberculosis* cultures (10 ml) were pelleted, resuspended in 1 ml RNAProBlue solution (Iepsa) and ribolysed for four 25 s cycles at 4.5 watt with the FastPrep-24 Instrument (MP Biomedicals). Cellular debris was removed by centrifugation at 18 400 × g for 15 min and a chloroform extraction performed prior to loading onto a Nucleospin RNA kit (Machery-Nagel) column. An on-column rDNase treatment was performed according to the manufacturer’s instructions and RNA was eluted in 30 μl nuclease free water. RNA quality and concentration were assessed using the Agilent Bioanalyzer RNA Nano Assay kit. RNA was DNase treated using the TURBO DNA-free Kit (Ambion). RNA (500 ng) was reverse transcribed using the Transcriptor first strand cDNA synthesis kit (Roche) according to the manufacturer’s instructions using 1 μM of reverse transcription primers (RT) indicated in Table B in S1 File. RT-qPCR was performed with the FastStart Essential DNA Green Master mix using 0.1 μM PCR primers and the LightCyler 96 machine (Roche). A standard curve was generated for each primer set using genomic DNA dilutions, and expression levels calculated relative to the reference gene, *sigA*.

### Enzyme and intracellular iron assays

Cultures for succinate dehydrogenase and aconitase assays were grown in 7H9 OADC. Log phase cultures were harvested (10 ml) and washed twice with PBS containing 0.05% Tween-80. Cell pellets were frozen at -80°C until analysis was performed. Pellets were resuspended in 1.5 ml cold 20 mM Tris-HCl buffer (pH 8.0) and cells ribolysed [42]. For succinate dehydrogenase assays cell free extracts were centrifuged at 2 700 × g for 10 min, while centrifugation was done at 21 130 × g for aconitase assays extracts. Succinate dehydrogenase assays were performed using a continuous assay as previously described [43]. Aconitase activity was measure using the Aconitase Assay kit (Sigma Aldrich). The total protein in each sample was determined using the BioRad protein assay. Activity was expressed relative to total protein content per sample.

The amount of intracellular iron was determined after three growth cycles in MM or MM + Fe^+3^. *M*. *tuberculosis* culture (20 ml) was harvested by centrifugation at 3 220 × g and cells washed once with PBS containing 0.05% Tween-80. Cells were resuspended in 1 ml 50 mM NaOH and ribolysed for three 30 s cycles at 5.0 watt with the FastPrep-24 Instrument (MP Biomedicals). Intracellular iron levels were measured using a previously described method [44]. The iron content in the lysate was standardised using the total protein content, as determined by the BioRad protein assay.

### Protein expression and purification

Rv1460 was heterologously expressed from the pETM-11Rv1460nss vector in *E*. *coli* Arctic express DE3 strain. This vector produces a N-terminally tagged Rv1460 protein that starts at position +73 relative to the Tuberculists annotation (i.e. re-annotated start site). The expression strain was cultured in LB broth at 30°C to an OD_600nm_ between 0.5 and 0.6. Protein production was then induced by the addition of 0.4 mM isopropyl β-D-thiogalactoside (IPTG) and allowed to proceed at 13°C for 24 h. Cells (1 L culture) were harvested and stored at -80°C. Frozen cells were re-suspended in lysis buffer (20 mM Tris-HCl, 500 mM NaCl, 20 mM imidazole, 1% glycerol, 1% dithiothreitol (DTT), 1 mg/ml lysozyme, pH 8.0) (50 ml) and incubated on ice for 30 min. Cells were lysed by sonication on ice using a QSonica probe sonicator (3/4 tip diameter) for 4 min at 50 watt with 15 s on and off intervals and the lysate clarified by centrifugation at 14 000 × g for 30 min at 4°C. The protein lysate (50 ml) was filtered through a 0.45 micron surfactant free cellulose acetate filter (Merck), before loading onto a HiTrap 5 ml nickel-NTA (Ni-NTA) column (GE Healthcare Life Sciences) coupled to an AKTA protein purification system (GE Healthcare Life Sciences) which had been equilibrated with 10 column volumes (CV) wash buffer (20 mM Tris-HCl, 500 mM NaCl, 20 mM imidazole, pH 8.0) at a flow rate of 5 ml per min. The column was washed with 10 CV of wash buffer and 5 ml flow through fractions collected. Protein was eluted with an imidazole gradient from 100% wash buffer to 70% elution buffer (20 mM Tris, 500 mM NaCl, 500 mM imidazole, pH 8.0) in 100 ml and 2.5 ml fractions collected. The remaining protein was eluted with 100% elution buffer. Protein elution was monitored at 280 nm. Fractions containing Rv1460 were pooled, DTT added to a final concentration of 1 mM and proteins concentrated in a volume of 5 ml. 6×His-tag cleavage with Tobacco Etch Virus protease (TEV) was performed concurrently with dialysis against 20 mM Tris-HCl, 150 mM NaCl, 1 mM DTT, 10% glycerol, pH 8.0. The dialysed sample was then passed over a Ni-NTA column to bind any uncleaved protein and the 6×His-tagged TEV. Fractions containing Rv1460 were pooled, concentrated and loaded onto a Sephadex S200 16/60 column equilibrated with gel filtration buffer (20 mM Tris-HCl, 150 mM NaCl, 10% glycerol, 1 mM DTT, pH 8.0). The fractions containing Rv1460 were pooled, concentrated and flash frozen.

The *E*. *coli* cysteine desulphurase, IscS_*E*.*coli*_, and scaffold protein, IscU_*E*.*coli*_, and TEV were expressed and purified as previously described [45,46] for use in the Fe-S cluster reconstitution assays. The *Azotobacter vinelandii* cysteine desulphurase, NifS, was expressed from the pDB845 vector in the BL21(DE3)pLysS strain (Table A in S1 File). The expression strain was cultured in LB broth at 37°C to an OD_600nm_ of between 0.7 and 0.9. Pyridoxal 5-phosphate (100 μM) was then added to the culture and protein expression induced by addition of 0.4 mM IPTG at 30°C for 2 hours. Protein purification was done as for Rv1460, with the following exceptions: the 7×His-tag of NifS was not cleaved, NifS gel filtration buffer contained only 1% glycerol and all buffers contained 0.5 mM Tris(2-carboxyethyl)phosphine (TCEP) instead of 1 mM DTT. Protein concentrations were determined by measuring absorbance at 280 nm on a Spectro UV-16 spectrophotometer (mrc).

### Fe-S cluster reconstitution assays

Enzymatic Fe-S reconstitution was done using the *E*. *coli* IscS_*E*.*coli*_ and IscU_*E*.*coli*_ proteins as previously described [47,48]. All reconstitution reactions were done in an anaerobic glove box (Bactron) in a 1 ml sealable quartz cuvette (Hellma Analytics, Jena, Germany). Reaction mixtures contained Rv1460 (50 μM), (NH_4_)_2_Fe(SO_4_)_2_ (25 μM), DTT (3 mM), IscS_*E*.*coli*_ (1 μM) and IscU_*E*.*coli*_ (5 μM), in 20 mM Tris-HCl, 150 mM NaCl, pH 8.0 in a total volume of 800 μl. The reaction was initiated by addition of 250 μM cysteine and monitored by recording UV-visible scans (250–600 nm) using an Ocean optics USB4000 spectrophotometer at regular intervals.

Enzymatic Fe-S cluster reconstitution was also done using the *A*. *vinelandii*, cysteine desulphurase, NifS, by modifying a previously described method [49]. Reaction mixtures contained Rv1460 (50 μM), DTT (3 mM), (NH_4_)_2_Fe(SO_4_)_2_ (25 μM), NifS (225 μM) and cysteine (500 μM) and were allowed to proceed for 6 hours before addition of additional (NH_4_)_2_Fe(SO_4_)_2_ to a final concentration of 50 μM. The reaction was allowed to proceed overnight and no buffer exchange was done before CD measurements.

Chemical reconstitution using lithium sulphide was performed as previously described [50]. Briefly, Rv1460 (50 μM) was reduced for an hour in buffer (20 mM Tris-HCl, 150 mM NaCl, pH 8.0) containing DTT (5 mM), where after the reaction was initiated by addition of a fivefold molar excess of iron ((NH_4_)_2_Fe(SO_4_)_2_ (250 μM)) and Li_2_S (250 μM). The reactions was allowed to continue for 2 hours. As an indication of the background reaction occurring in the absence of Rv1460 protein, the Rv1460 protein was omitted. Unbound species were separated from Rv1460 by buffer exchange using PD Minitrap G-25 columns (GE Healthcare) pre-equilibrated with Rv1460 gel filtration buffer according to the manufacturer’s instructions. Fractions (~250 μl) were collected and those that contained the Rv1460 protein were pooled (~750 μl). The concentration of protein was determined using the Direct Detect Infrared Spectrometer (Merck) according to the manufacturer’s instructions.

### Circular dichroism

Circular dichroism (CD) measurements were done on a Chirascan-plus CD spectrometer fitted with a cell holder thermostatted by a Single cell Peltier Temperature controller (containing a Quantum North West TC125 Unit) at the Central Analytical Facility of Stellenbosch University NMR unit. Far-UV and near-UV CD spectra were recorded using fused silica cuvettes (Hellma Analytics, Jena, Germany) with path lengths of 1 mm and 10 mm, respectively at 20°C (using a 2 nm bandwidth 0.25 s per set with adaptive sampling enabled). Data was smoothed fivefold and tenfold respectively for the far- and near-UV CD using the Chirascan-plus software. Protein concentrations were 1.27 mg/ml for near-UV and 0.12 mg/ml for far-UV CD.

Thermal unfolding curves were obtained by monitoring the ellipticity (θ) at 222 nm in a 1 mm path length sealed cuvette, every 2°C at a heating rate of 2°C/min from 5°C to 91°C at a bandwidth of 2 nm (timebase 2 s, points 20, samples 4000, time per reading 25 us). The 20 readings taken over time per wavelength were averaged. CD measurements are presented as the ellipticity (θ) or molar CD extinction coefficient (Δε_M_) for near-UV and mean residue CD coefficient (Δε_MRW_) for far-UV CD measurements.

### Statistical analysis

All statistical analyses were performed using GraphPad Prism Software version 6.05 and statistical analysis language R version 3.4.1.

## Results

### Rv1460 is a SufR homologue that is highly conserved in mycobacteria

**Fig 1:**
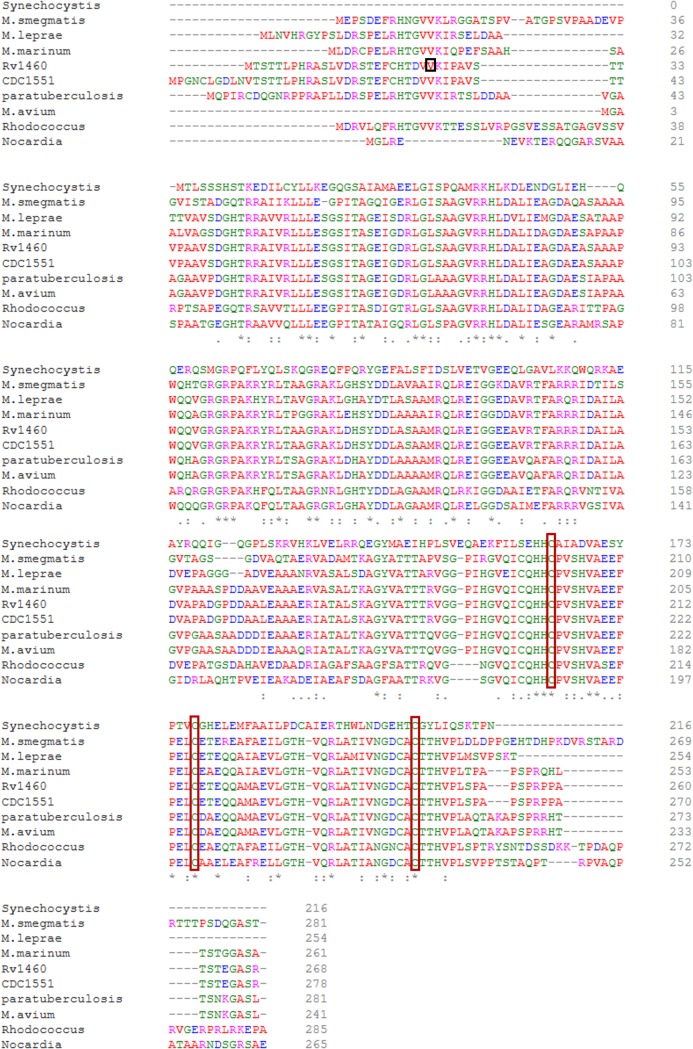
Rv1460 is a SufR homologue. Maestro Multiple Sequence Viewer multiple alignment of Rv1460 [ACJ83238] with homologues in selected mycobacteria and SufR homologues present in other organisms. The re-annotated start site of Rv1460 is indicated by the red box. Conserved cysteine residues are indicated by the orange boxes. *Mycobacterium smegmatis*, transcriptional regulator [WP_011728830]; *Mycobacterium leprae*, transcriptional regulator [WP_010907825]; *Mycobacterium marinum* E11, transcriptional regulatory protein [CDM76334]; *Mycobacterium tuberculosis* CDC1551, conserved hypothetical protein [AAK45771]; *Mycobacterium avium* subsp. paratuberculosis K-10, hypothetical protein MAP_1186 [AAS03503]; *Mycobacterium avium* 104, DNA-binding protein [ABK68668]; *Rhodococcus fascians*, transcriptional regulator [WP_037190040]; *Nocardia veteran*, transcriptional regulator [WP_051031599] and *Synechocystis* sp. PCC 6803, SufR [WP 020862050].

Alignment of the amino acid sequence of the protein encoded by *Rv1460* with homologues from other mycobacterial species revealed that the protein is highly conserved in both pathogenic and non-pathogenic mycobacteria (Fig 1). In particular, the three cysteine residues at positions 203, 216 and 244, predicted to provide ligands to coordinate an Fe-S cluster [35], are conserved in all the homologues analysed. InterProScan identified a winged helix-turn-helix DNA-binding motif (IPR011991), in the C-terminal region of Rv1460, spanning amino acids 42 to 150. Comparison with SufR homologues from Synechocystis, Rhodococcus and Nocardia revealed that most of the mycobacterial homologues possess an N-terminal extension not present in other species. The extension varied in both length and sequence in the mycobacterial homologues (Fig 1).

### Characterisation of the *Rv1460* promoter

**Fig 2:**
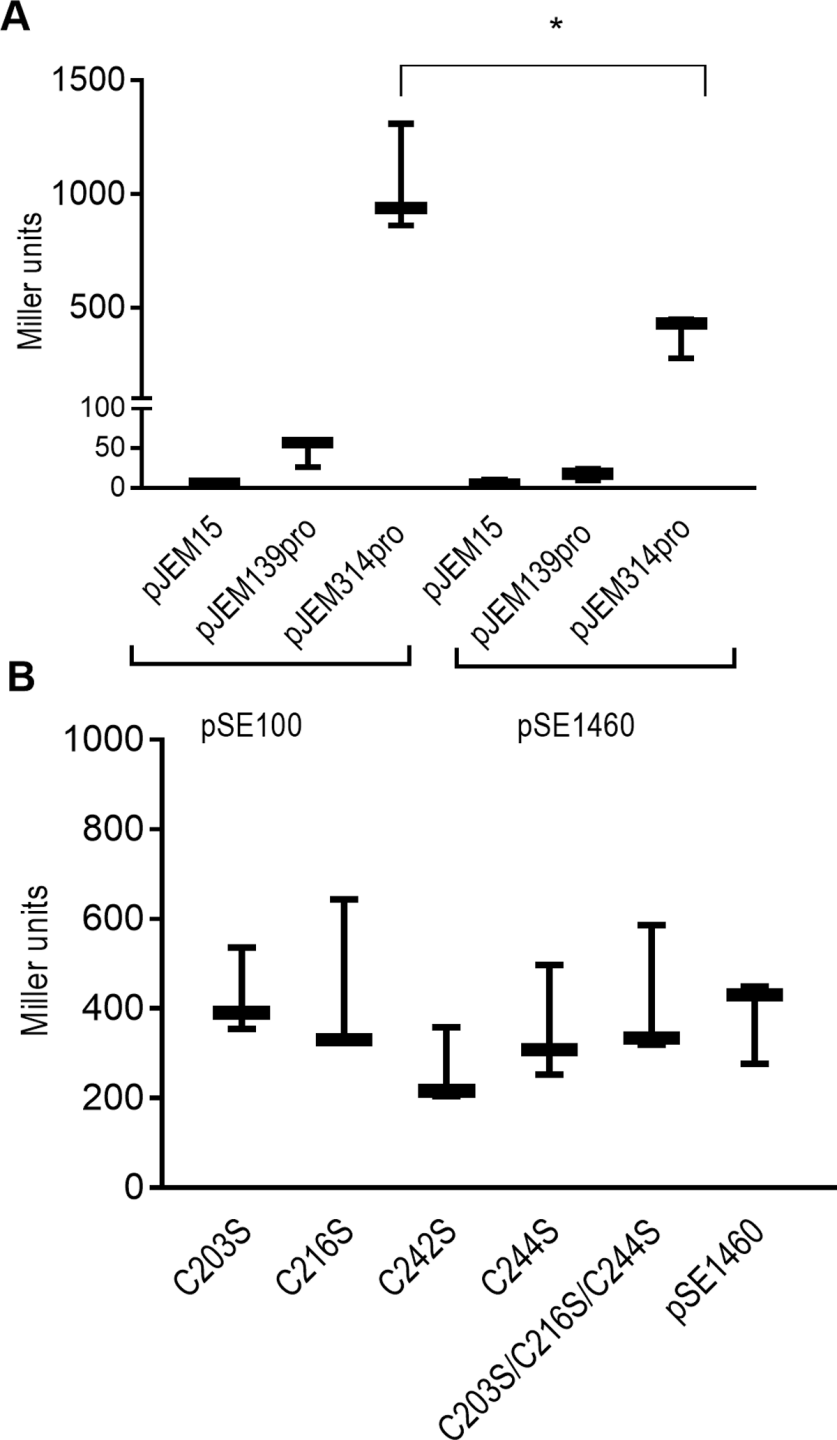
Rv1460 represses its own expression. (A) Schematic representation of the β-galactosidase assay procedure. Each reporter vector (pJEM15, pJEM139pro or pJEM314pro) (containing the 139 bp or 314 bp regions upstream of *Rv1460* fused to a *lacZ* reporter gene) was co-transformed into *M*. *smegmatis* with a protein expression vector either encoding no protein (pSE100) or encoding Rv1460 (indicated by the grey oval). (B) β-galactosidase activity from the 139 bp and 314 bp promoter fragments with (pSE1460) and without (pSE100) co-expression of Rv1460 in *M*. *smegmatis*. Transcriptional repression by wild-type or variants of Rv1460 results in a decrease in β-galactosidase activity, which is expressed in Miller units calculated as follows: 200 × (change in OD_450nm_) per mg protein per min. The results shown are the mean and standard deviation for three experiments. Statistical analysis compared the mean activity for each plasmid with or without co-expression of Rv1460 e.g. pJEM15 (pSE100) vs. pJEM15 (pSE1460) using an unpaired t-test (*p ≤0.05). (C) β-galactosidase activity from the 314 bp promoter (pJEM314pro) when wild-type (pSE1460) or mutated forms of Rv1460 (pSE_C203S or pSE_C216S or pSE_C242S or pSE_C244S or pSE_C203S/C216S/C244S) are expressed in *M*. *smegmatis*. The results shown are the mean and standard deviation for three experiments. Statistical analysis compared the mean activity for wild-type with each variant using an unpaired t-test.

Genome-wide transcription start-site mapping re-annotated the transcriptional start site of Rv1460 at +73, and identified a 120 bp 5´ untranslated region [51]. CHiP-seq analysis identified a binding site for Rv1460 20 bp upstream of this +73 start codon [52]. The structure of the promoter region of *Rv1460* was therefore investigated using a β-galactosidase reporter assay. A region 139 bp upstream of the start site annotated in Tuberculist was amplified and cloned upstream of a promoterless *lacZ* gene in the pJEM15 vector to generate the pJEM139pro reporter vector. A second 314 bp region, which included the 139 bp upstream region, and two alternate start codons (positions +73 and +142) was also cloned into pJEM15 to generate pJEM314pro. Expression from the 139 bp region was only slightly above baseline, while expression from the 314 bp promoter region was 200-fold higher than baseline (Fig 2B), suggesting that the promoter is located downstream of the Tuberculist annotated start site. Transcriptional start site mapping was performed and confirmed the start site at +73, but found no evidence to support a start site at +142 or for a 5’ untranslated region (Figure A, panel I in S1 File).

### Rv1460 is a transcriptional repressor

ChiP-seq identified a binding site for Rv1460 20 bp upstream of the +73 start codon, suggesting that it regulates the expression of *Rv1460* through direct interaction with the promoter region of the gene [52]. To investigate this, Rv1460 was co-expressed from the protein expression vector, pSE1460, with each of the reporter vectors (pJEM15, pJEM314pro or pJEM139pro) in *M*. *smegmatis* (Fig 2A). Co-expression of Rv1460 with pJEM314pro resulted in a threefold decrease in β-galactosidase activity relative to the strain without Rv1460 (i.e. pSE100 and pJEM314pro) (Fig 2B). This confirms that Rv1460 represses its own expression.

**Fig 3:**
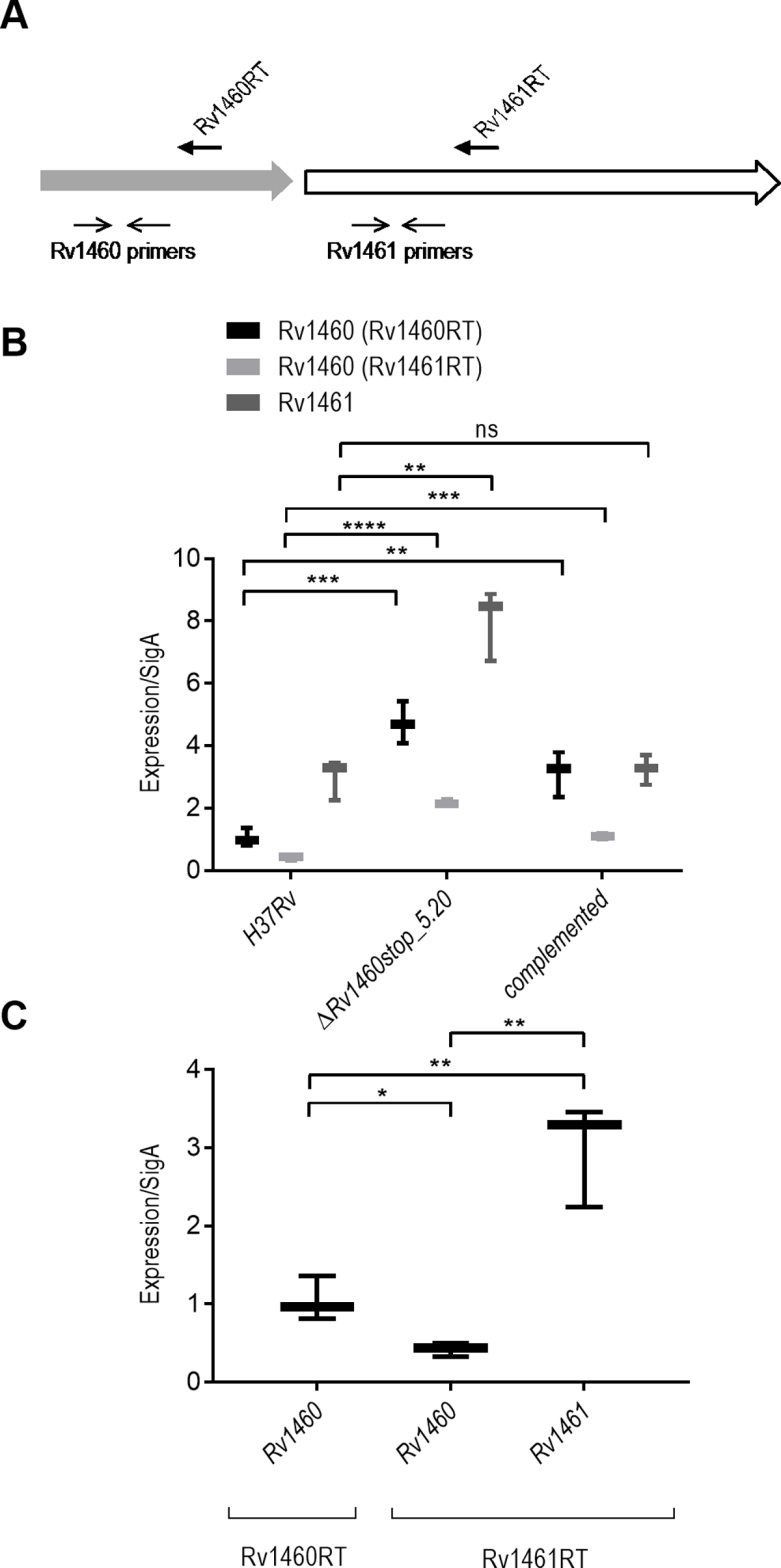
Rv1460 is a repressor of the *suf* operon. (A) Location of RT and gene specific primers used for RT-qPCR (Table B in S1 File). (B) Relative gene expression in H37Rv (wild-type), Δ*Rv1460*stop_5.20 mutant and complemented strain. (C) Relative gene expression using the Rv1460RT and Rv1461RT primers and gene specific *Rv1460* and *Rv1461* primers in H37Rv. The results shown are the mean and standard deviation of three experiments and stars indicate significant differences determined by unpaired t-test: * p≤0.05, ** p ≤0.01, ***p≤0.001**** p ≤0.0001.

The role of Rv1460 as a repressor in *M*. *tuberculosis* was then investigated by introducing deletions in *Rv1460* which would render the protein non-functional. To do this, two allelic exchange substrates, aimed at introducing distinct deletions in *Rv1460*, were generated. The first substrate, namely p2NIL17Δ*Rv1460*, would generate an in-frame, un-marked deletion which would remove 175 of the 268 amino acids of the protein (codons 36 to 211 deleted according to the Tuberculist annotation). The second substrate, p2NIL17*Rv1460*stop, would remove the DNA-binding domain (codons 36 to 114 according to the Tuberculist annotation) and introduce a premature stop codon at position 122. Attempts to generate the Δ*Rv1460* mutant (deleting codons 36 to 211) by two-step allelic exchange were unsuccessful; however when a second copy of the *Rv1460* gene was present at the *attB* site in the chromosome, deletion of the native gene was readily achieved (Δ*Rv1460 attB*::pMV1460 strain) (Figure B in S1 File). PCR was used to confirm that both up- and downstream single cross overs (SCOs) were obtained, indicating efficient homologous recombination in both configurations without bias for either configuration (Table E in S1 File). *M*. *tuberculosis* Rv1460 truncation mutants (Δ*Rv1460*stop) were readily generated using the p2NIL17*Rv1460*stop construct, as confirmed by PCR and Southern blotting (Figure C in S1 File). Three independently isolated truncation mutants (Δ*Rv1460*stop_1.19, Δ*Rv1460*stop_5.19 and Δ*Rv1460*stop_5.20) were selected for further analysis. PCR amplification and Sanger sequencing of the region around *Rv1460* confirmed that the deletion introduced into these three strains was identical (Figure D in S1 File). The role of Rv1460 in regulating the expression of *Rv1460* was investigated by evaluating transcript levels in one of the truncation mutants (Δ*Rv1460*stop_5.20), and its complemented strain (Fig 3B; Rv1460(Rv1460RT)). Induction of *Rv1460* was observed in the Δ*Rv1460*stop_5.20 mutant and decreased in the complemented strain, confirming that Rv1460 functions as a repressor of its own expression in *M*. *tuberculosis* (Fig 3B).

### Regulation of the *suf* operon by Rv1460

Transcription of the *Rv1460-Rv1461-Rv1462-Rv1463-csd-Rv1465-Rv1466* gene cluster as an operon was previously demonstrated in *M*. *smegmatis*, *Mycobacterium bovis* and *M*. *tuberculosis*, however evidence to suggest that the *Rv1460* homologue in *M*. *smegmatis* can be transcribed independently also exists [16,52,53]. The transcription of *Rv1460* was therefore compared using two different RT primers in the H37Rv (wild-type) strain, namely, Rv1460RT, which binds within *Rv1460* and, Rv1461RT, which binds within *Rv1461* (Fig 3A and Table B in S1 File). The amount of *Rv1460* transcript detected using the Rv1460RT primer was 2.5-fold higher than when the Rv1461RT primer was used. This confirms that *Rv1460* is transcribed as part of the operon, but suggests that *Rv1460* may also be transcribed independently. When the expression of *Rv1460* and *Rv1461* was evaluated using the Rv1461RT primer, the *Rv1461* levels were 7-fold higher than *Rv1460* in the wild-type strain (Fig 3C), further supporting the idea that *Rv1460* and *Rv1461-Rv1462-Rv1463-csd-Rv1465-Rv1466* may be expressed from distinct promoters in *M*. *tuberculosis*. In agreement with this, a ribosomal binding site has been predicted upstream of *Rv1461* (Tuberculist). Transcriptional start site mapping was performed for *Rv1461* and identified a start site -17 bp from the start of the gene (Figure A, panel II in S1 File), supporting this hypothesis.

Expression of *Rv1461* was induced 2.7-fold in the truncation mutant, and restored to wild-type levels in the complemented strain, confirming that Rv1460 also functions as a repressor of *Rv1461*, and thus the *suf* operon [16,52,53]. Comparison of the *Rv1460* transcript level using either the Rv1460RT or the Rv1461RT primers in the complemented strain (Fig 3B) confirmed expression of the copy of *Rv1460* at the *attB* site, since *Rv1461* is not present at the *attB* site and the Rv1461RT primer thus detects transcription of *Rv1460* in its native genomic context, while the transcript levels detected using the Rv1460RT primer represents expression occurring from both sites.

### Truncation of Rv1460 is deleterious for the growth of *M*. *tuberculosis* under standard culture conditions

**Fig 4:**
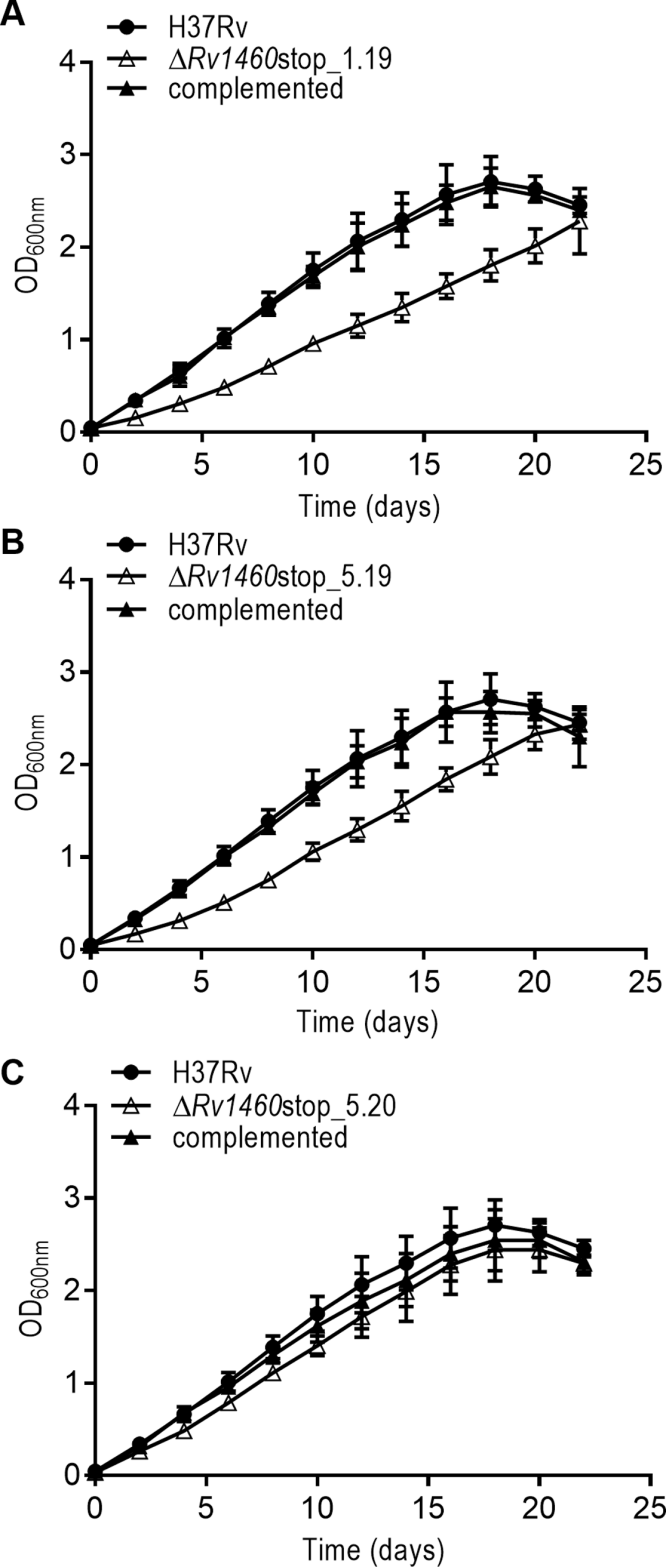
Rv1460 truncation mutants are impaired for growth in vitro under standard culture conditions. (A–C) Growth of H37Rv (wild-type), three truncation (Δ*Rv1460*stop) mutants and their complemented strains under standard conditions in 7H9 OADC. The results shown are the mean and standard deviation of three experiments.

The impact of the loss of Rv1460 and concomitant increase in *suf* operon expression on the growth of *M*. *tuberculosis* was determined by evaluating the growth of the three truncation mutants Δ*Rv1460*stop_1.19, Δ*Rv1460*stop_5.19 and Δ*Rv1460*stop_5.20 and their complemented strains. All three mutants displayed a growth defect under standard culture conditions in liquid (Fig 4) and on solid media (Figures E-F in S1 File), although the severity of the phenotype differed between strains. In liquid culture, the mutants displayed an extended lag phase, while the growth rate in exponential phase was similar to that of the wild-type strain. In all cases, the growth phenotype could be reversed by genetic complementation with *Rv1460* at the *attB* site (Fig 4).

**Fig 5:**
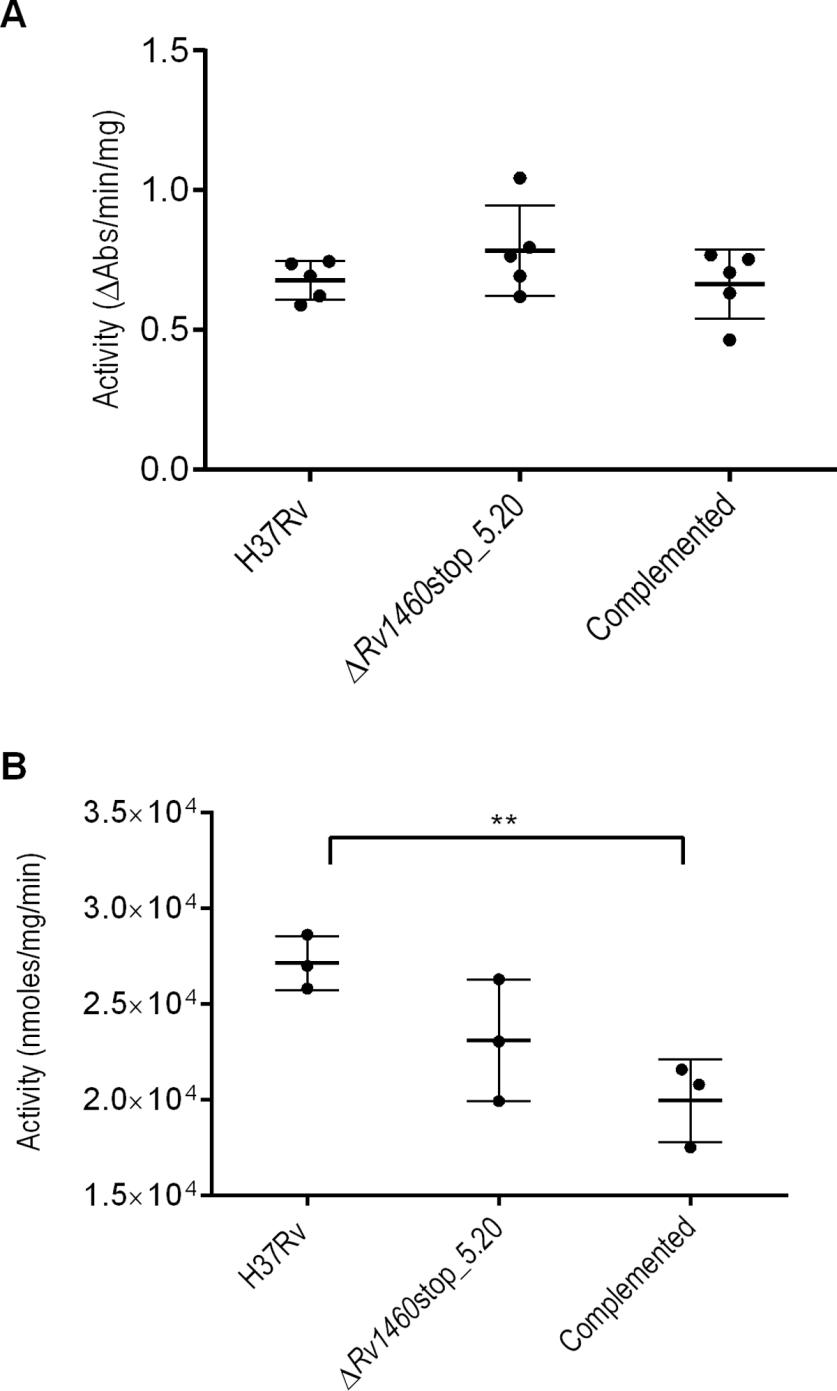
Succinate dehydrogenase and aconitase activity is not impaired in Rv1460 truncation mutants. (A) Succinate dehydrogenase activity and (B) aconitase activity in the H37Rv (wild-type), Δ*Rv1460*stop_5.20 and complemented strains cultured in 7H9 OADC. Activity was standardised relative to total protein. The results shown are the mean and standard deviation of five and three experiments respectively.

The impact of *suf* operon induction on Fe-S cluster containing enzymes was investigated by measuring succinate dehydrogenase and aconitase activity in the wild-type, Δ*Rv1460*stop_5.20 mutant and complemented strains (Fig 5). No significant difference in the succinate dehydrogenase activity was observed between the three strains. In addition, no significant difference in the aconitase activity was observed between the wild-type and mutant strain, while the aconitase activity in the complemented strain was 1.36-fold lower than the wild-type strain (p = 0.0085) (Fig 5).

### Truncation of Rv1460 in *M*. *tuberculosis* does not impair growth under iron limiting conditions

**Fig 6:**
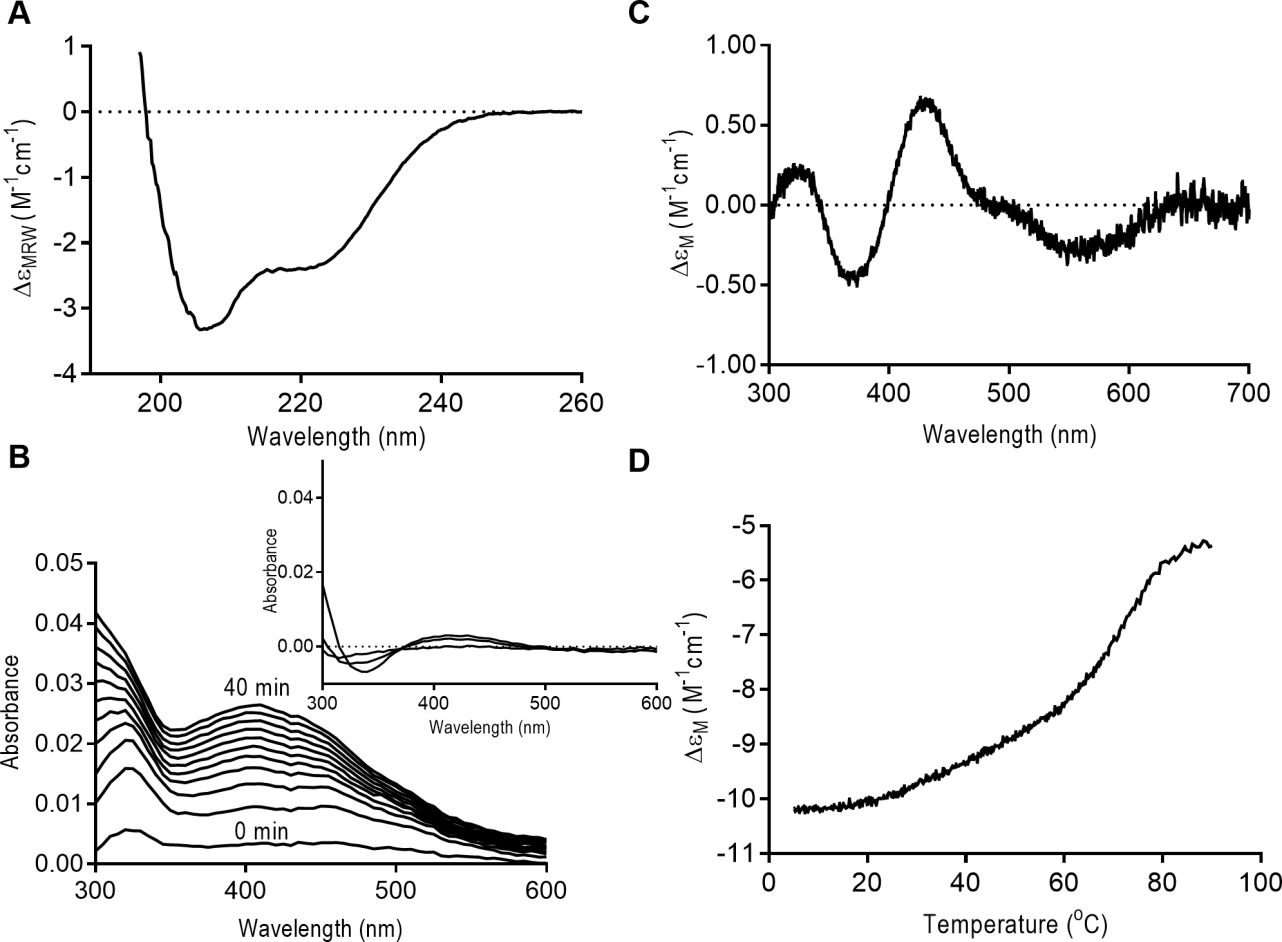
Rv1460 truncation mutants are not impaired for growth under iron-limiting conditions. Growth of H37Rv (wild-type), three truncation mutants (Δ*Rv1460*stop) and complemented strains in (A–C) MM + Fe^+3^ and (D–F) MM for three growth cycles (subcultured on day 14 to an OD_600nm_ of 0.05). The results shown are the mean and standard deviation of three experiments.

Induction of the *suf* operon was previously observed during iron limitation [54]. The growth of the three truncation mutants was therefore investigated (Δ*Rv1460*stop_1.19, Δ*Rv1460*stop_5.19 and Δ*Rv1460*stop_5.20) at two iron concentrations (~2 μM and 50 μM FeCl_3_). The strains were cultured for three growth cycles to deplete intracellular iron stores (Fig 6). Decreasing the iron in the media resulted in a concomitant decrease in the optical density at which cultures entered stationary phase, while an increase in the length of the lag phase was observed with subsequent growth cycles. No difference in growth was observed between the wild-type and mutant strains. The intracellular iron content of the strains was determined after the third growth cycle. No difference in the intracellular iron levels was observed between the wild-type, mutant and complemented strains, while in all cases intracellular iron levels were lower when only residual iron (~2 μM FeCl_3_) was present in the media (Table F in S1 File).

### C203, C216 and C244, but not C242 are required for Rv1460 function in *M*. *tuberculosis*

The importance of Rv1460 in *M*. *tuberculosis* was explored further by trying to remove the pMV1460 vector from the *attB* site in the complemented deletion mutant (i.e. Δ*Rv1460 attB*::pMV1460). The pMV1460 vector confers hygromycin resistance to the Δ*Rv1460 attB*::pMV1460 strain. The *M*. *tuberculosis* Δ*Rv1460 attB*::pMV1460 strain was transformed with either empty vector, pCV-125 (integrating vector containing a kanamycin resistance gene), or pCV-125 containing a wild-type copy of *Rv1460* (pCV1460). Transformation with the empty vector resulted in approximately 1000-fold fewer kanamycin resistant transformants as compared to the vector expressing the wild-type copy of *Rv1460* (Table 1). Colonies resulting from transformation with the pCV1460 vector were unable to grow on hygromycin. The absence of the hygromycin resistance gene and presence of the kanamycin resistance gene (*aph*) was verified by PCR (Figure G in S1 File), confirming that pMV1460 (conferring hygromycin resistance) was replaced by pCV1460 (conferring kanamycin resistance) at the *attB* site. Approximately 60% of the empty vector pCV-125 transformants were unable to grow on plates containing hygromycin, and replacement of pMV1460 by pCV-125 in these transformants was confirmed by PCR (Figure G in S1 File). The low frequency at which the Δ*Rv1460 attB*::pCV-125 strain could be recovered suggests that replacement with the empty vector and thus the loss of Rv1460 was deleterious.

**Table 1:** Number of transformants recovered when complementation vector in *M*. *tuberculosis* Δ*Rv1460 attB*::pMV1460 was swopped with vector indicated.

Vector	Transformants^a^		
	E1	E2	E3
pCV1460	5.7 × 10^5^	1.1 × 10^5^	3.2 × 10^5^
pCV-125	1.7 × 10^2^	7.2 × 10^2^	3.6 × 10^2^
pCV1460_C203S	4.5 × 10^2^	2.4 × 10^2^	9.6 × 10^1^
pCV1460_C216S	3.2 × 10^2^	1.6 × 10^2^	1.2 × 10^2^
pCV1460_C244S	5.5 × 10^2^	5.9 × 10^2^	1.4 × 10^2^
pCV1460_C203S/C216S/C244S	1.8 × 10^2^	1.9 × 10^2^	1.4 × 10^2^
pCV1460_C242S	7.8 × 10^5^	2.3 × 10^5^	1.2 × 10^5^

^a^Data for three independent experiments (E1-E3)

Three conserved cysteine residues have been predicted to be involved in coordinating an Fe-S cluster in Rv1460. It was reasoned that the essentiality of the three conserved cysteine residues (C203, C216, C244) in Rv1460 could be evaluated by comparing the efficiency with which vectors encoding variants of Rv1460 were able to replace the pMV1460 vector in the *M*. *tuberculosis* Δ*Rv1460 attB*::pMV1460 strain. The Δ*Rv1460 attB*::pMV1460 strain was transformed with pCV1460 derivative vectors expressing either Rv1460_C203S, Rv1460_C216S, Rv1460_C244S or Rv1460_C203S/C216S/C244S (Table C in S1 File), and transformants recovered on kanamycin plates. The number of transformants recovered in each case was comparable to those observed with the empty vector, suggesting that these residues are required for the function of Rv1460 (Table 1). As was observed for the empty vector (pCV-125), approximately 40% of these transformants grew on plates containing both kanamycin and hygromycin indicating that the pMV1460 vector was not replaced with the pCV-125 vectors expressing the serine residue variants. In contrast, transformation with a vector expressing Rv1460_C242S, a variant of a cysteine not predicted to coordinate the Fe-S cluster, displayed the same transformation efficiency as the pMV1460 vector expressing the wild-type protein (Table 1). None of these transformants grew on plates containing hygromycin suggesting that the pMV1460 vector had been replaced. Therefore, the cysteine at position 242 is not essential for Rv1460 function.

The inability of the serine variants to complement the Δ*Rv1460* mutant may be due to altered binding of the variants to the *Rv1460* promoter. The ability of the Rv1460 variants to repress expression from the *Rv1460* promoter was therefore investigated using the β-galactosidase promoter activity assays in *M*. *smegmatis*. Transcriptional repression by the Rv1460_C203S, Rv1460_C216S, Rv1460_C242S and Rv1460_C244S variants was similar to that observed for wild-type Rv1460, suggesting that they are still able to bind to the *Rv1460* promoter (Fig 2C).

### Rv1460 binds an Fe-S cluster

Since SufR coordinates an Fe-S cluster, we investigated the ability of Rv1460 to coordinate an Fe-S cluster in vitro. To do this, recombinant Rv1460 was expressed in *E*. *coli* and purified under aerobic conditions by Ni-NTA and gel filtration chromatography. Elution of the recombinant protein from gel filtration occurred as a broad peak between the 158 kDa and 17 kDa standards (Figure H, part A in S1 File). Analysis of the gel filtration fractions by SDS-PAGE revealed a band which corresponds to the size of the monomeric form of the protein (Figure H, part B in S1 File). The far-UV CD spectrum of the purified protein indicated that Rv1460 is folded and has a mixed alpha-beta conformation (Figure J, part A in S1 File). The UV-visible absorbance spectrum of purified recombinant Rv1460 showed that no Fe-S cluster was bound to the protein (data not shown), presumably due to exposure to oxygen during the purification.

A method to reconstitute Fe-S clusters enzymatically in vitro was previously developed using the *E*. *coli* cysteine desulphurase, IscS_*E*.*coli*_, and scaffold protein, IscU_*E*.*coli*_ [48,55]. The ability of this system to reconstitute an Fe-S cluster on purified Rv1460 under anaerobic conditions was investigated. *E*. *coli* IscS_*E*.*coli*_ and IscU_*E*.*coli*_ were purified as previously described [45]. The reconstitution reaction contained cysteine, iron and DTT, and was monitored by UV-visible spectroscopy over time. Rv1460 was present at 50 μM, while catalytic amounts of IscS (1 μM) and IscU (5 μM) were added. Since the high DTT concentration in the reaction can generate other Fe-S species that absorb in the 420 nm range [56], a reaction without Rv1460 protein was included as a control. In the reaction containing Rv1460 there was a small increase in the absorbance between 400 and 500 nm with peaks at 420 nm and 450 nm, which transitioned to a single peak at 420 nm (Figure I, part A in S1 File). The near-UV CD spectrum for this reaction after the reconstitution displayed two maxima at 330 nm and 420 nm, supporting the presence of a 2Fe– 2S cluster (Figure I, part C in S1 File). However, the control reaction without Rv1460 also showed an increase in the UV-visible absorbance spectrum between 400 nm and 500 nm (Figure I, part B in S1 File), and maxima at 330 nm and 420 nm in the near-UV CD spectrum (albeit not as large as the Rv1460 containing reaction) (Figure I, part C in S1 File). This method could therefore not be used to conclusively confirm that the 2Fe-2S clusters being formed are bound to Rv1460.

**Fig 7:**
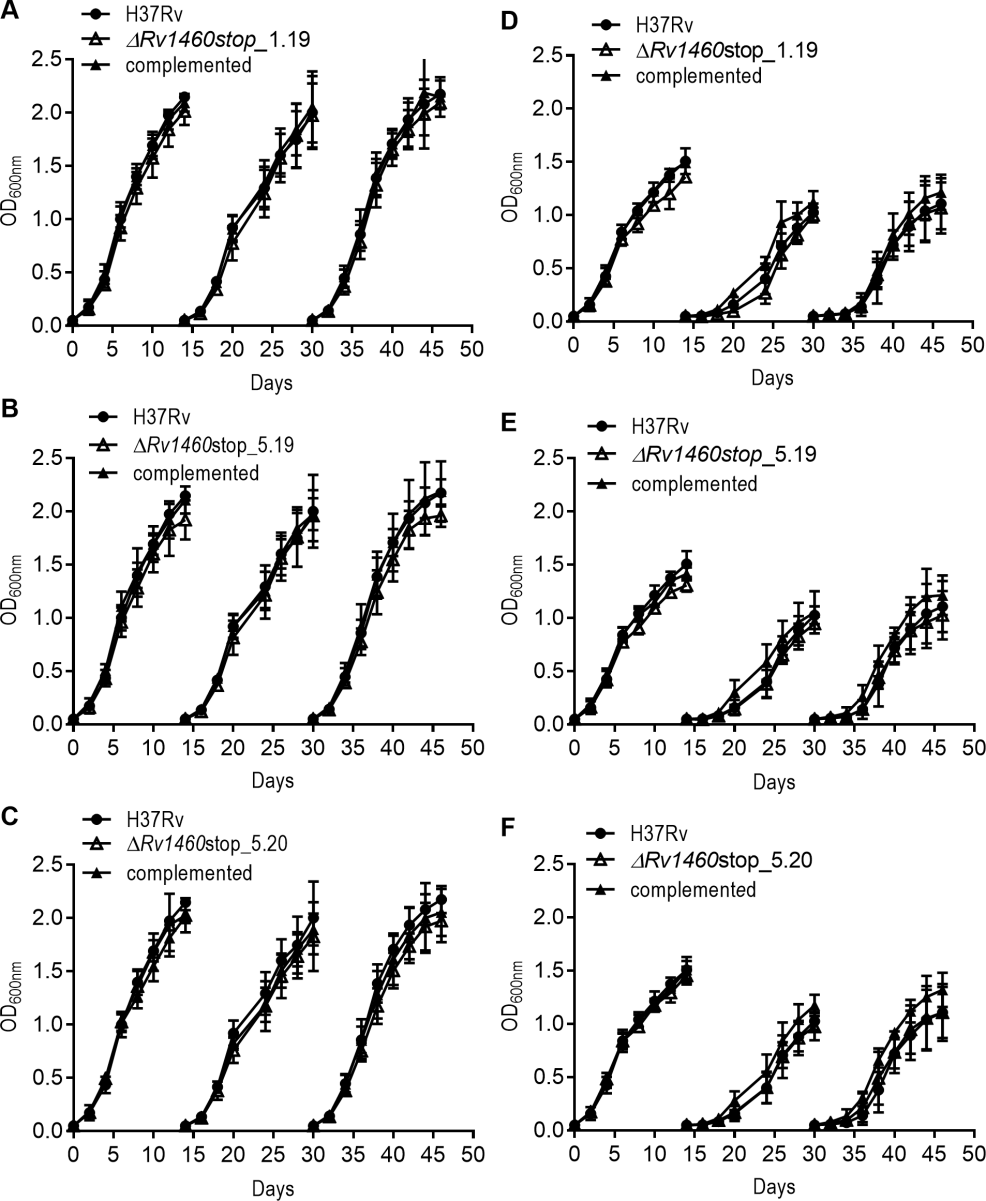
Rv1460 binds an Fe-S cluster. (A) UV-visible spectrum of NifS reconstitution reaction (at 1 and 20 hours) containing Rv1460 (50 μM) (black) or without Rv1460 (grey). The absorbance at 2 minutes was used as a blank for the reaction. (B) Near-UV CD spectrum of NifS reconstitution reaction containing Rv1460 (50 μM). Insert shows the near-UV CD spectrum (Ellipticity in millidegrees) of reactions with (solid line) and without Rv1460 (dashed line). (C) UV-visible spectrum of lithium sulphide reconstitution reaction with (black) and without Rv1460 (50 μM) over time. The absorbance at 2 minutes was used as a blank for the reaction. UV-visible spectrum of the reactions after buffer exchange are indicated in Figure K. in S1 File (D) Near-UV CD spectrum of lithium sulphide reconstitution reaction after buffer exchange containing (23 μM) Rv1460. Insert shows the near-UV CD (Ellipticity in millidegrees) of reactions with (solid line) and without Rv1460 (dashed line) after buffer exchange.

Enzymatic reconstitution of *M*. *tuberculosis* WhiB proteins was previously performed with the *A*. *vinelandii* cysteine desulphurase, NifS, which is able to form clusters directly on apo-proteins without a scaffold protein [49]. To investigate if NifS could reconstitute a cluster on Rv1460, a reconstitution reaction with purified NifS containing cysteine, iron and DTT was set up with or without Rv1460 (50 μM). An increase in absorbance around 420 nm was observed over several hours for both reactions, although the shape of the two curves was different (Fig 7A). The near-UV CD spectrum of the reaction with Rv1460 showed a peak at 330 nm and 420 nm (Fig 7B). These peaks are characteristic of the formation of a 2Fe-2S cluster and were absent in the reaction without Rv1460 (Fig 7B insert). The increase in absorbance in the reaction without Rv1460 therefore appears to be from the formation of alternative species due to the high DTT concentration, as previously reported [56].

Chemical reconstitution, in which an excess of iron and sulphide drive the spontaneous formation of Fe-S clusters, is a method commonly used for reconstitutions [50]. To further verify the co-ordination of an Fe-S cluster by Rv1460, chemical reconstitution using lithium sulphide was performed. Reactions contained a five-fold molar excess of lithium sulphide and iron, 5mM DTT, with 50 μM Rv1460. A control reaction without Rv1460 was also included (without Rv1460). In the reaction containing Rv1460, an increase in absorbance was observed between 300 nm and 500 nm for the first 120 min, after which the reading plateaued (Fig 7C). A similar increase was observed for the first 20 min in the control reaction not containing Rv1460, although the shape of the curve was different (Fig 7C). Following buffer exchange to remove species which are not protein-bound, the UV-visible spectrum of Rv1460 showed a peak at 420 nm (Figure K in S1 File). The near-UV CD spectrum was measure for both reactions and the reaction containing Rv1460 showed a spectrum characteristic of a 2Fe-2S cluster with peaks at 330 nm and 420 nm (Fig 7D) while the control reaction without Rv1460 did not (Fig 7D insert), confirming that the cluster is bound to Rv1460.

The far-UV CD spectrums for apo- and holo-Rv1460 were comparable, showing a mixed alpha-beta conformation (Figure J, part A in S1 File). This suggests that Rv1460 does not require its Fe-S cluster for proper folding. Thermal denaturation of apo- and holo-Rv1460 was monitored by far-UV CD at 222 nm between 5 and 91°C (Figure J, part B in S1 File). The thermal unfolding profile was complex and similar for the apo-and holo-Rv1460, which suggests binding of the Fe-S cluster to Rv1460 does not significantly alter protein stability.

## Discussion

Optimal expression of Fe-S cluster biogenesis systems is essential as excessive expression is toxic and inhibits bacterial growth [11], while inadequate expression would have a deleterious effect on Fe-S cluster containing enzymes and regulators. These systems are often regulated by proteins that themselves contain Fe-S clusters, as they are able to modulate expression in response to the changing demand for clusters within the cell. In this study, we characterised the transcriptional regulator, a SufR homologue, encoded by the first gene in the *suf* operon, *Rv1460*, and investigated its role as a regulator of the *suf* operon in *M*. *tuberculosis*.

**Fig 8:**
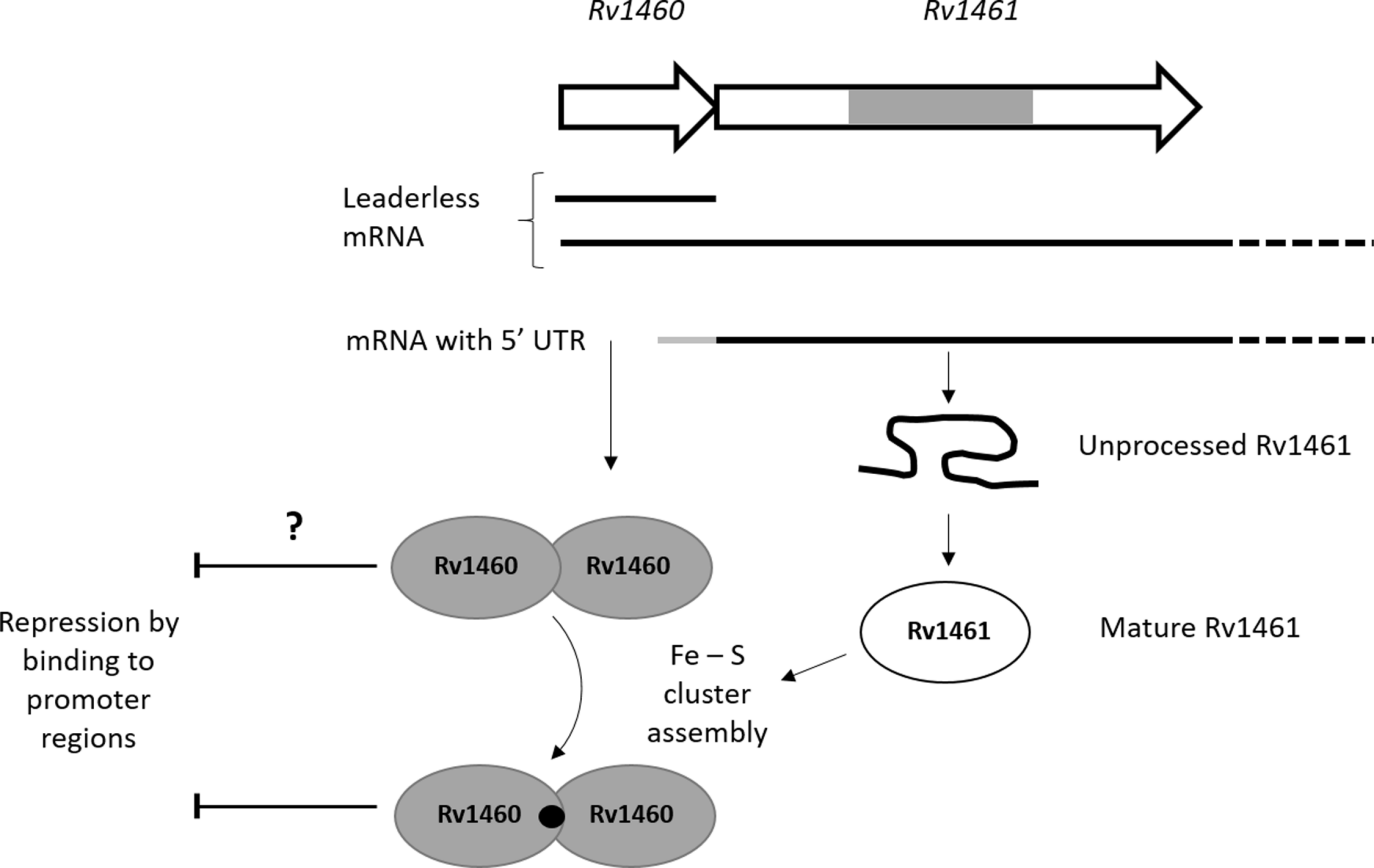
Fe-S cluster assembly is regulated by multiple mechanisms in *M*. *tuberculosis*. *Rv1460* is transcribed independently from the operon allowing differential expression and regulation by the repressor. Initiation of translation is leaderless upstream of *Rv1460*, while a 5’-UTR containing a ribosome binding site regulates translation of *Rv1461*. The intein within Rv1461 must be resolved before it is functional and may represent an additional level of regulation of the system. Rv1460 is predicted to bind within the promoter region upstream of Rv1460 as well as within Rv1461 (as indicated by the dashed line boxes). Binding sites for other regulators within the Rv1460 promoter region and within the operon are also predicted, providing another level of regulation. Binding of an Fe-S cluster may change Rv1460’s affinity for DNA. Genes are not drawn to scale. The association of the Fe-S cluster machinery is inferred from other bacterial SUF systems, but has not been experimentally validated in mycobacteria. Cysteine is provided by the cysteine desulphurase, while the source of iron is unknown. The oligomeric state of Rv1460 is not indicated since it has not been confirmed, and the ratio of Fe-S cluster to Rv1460 protein is unknown.

Transcriptional start site mapping revealed that *Rv1460* is transcribed from position +73 relative to the Tuberculist annotation of the gene (Figure A in S1 File). This corresponds to the start-site identified by genome-wide transcriptional mapping [51], and suggests that *Rv1460* is expressed as a leaderless transcript. RT-qPCR analysis suggested that *Rv1460* is transcribed as part of the operon, as well as independently (Fig 3). The identification of a transcriptional start site for *Rv1461*–17 bp from the start of the gene (Figure A in S1 File) supports this hypothesis, although we cannot rule out the possibility that this mRNA fragment originates from degradation of the longer transcript. Leaderless translational initiation occurs much more frequently in mycobacteria than *E*. *coli*, and genes which contain a 5´-untranslated region (UTR) are hypothesized to be subjected to additional regulation, by virtue of the sequence of a ribosome binding site or secondary structure in the UTR [51]. The leaderless translational initiation of Rv1460 and the leadered initiation of Rv1461 may therefore represent a mechanism of regulating the level of Rv1460 protein independently of the operon. Fig 8 shows a proposed model of the levels of regulation which may impact the production of *suf* operon-encoded proteins.

In other organisms harbouring a *suf* operon, SufR is often divergently transcribed or located at a different position in the genome [34], allowing for the independent regulation of the repressor. In Synechocystis, a high affinity SufR binding site controls the expression of *sufBCDS*, while a low affinity binding site controls the expression of *sufR* itself [35]. In *E*. *coli*, SufR is absent, and induction of the *sufABCDSE* operon is mediated by several regulators, including the regulator of the ISC system, IscR. Conversion of the Fe-S cluster containing regulator IscR to its apo-form results in release from the type 1 motif upstream of the *iscRSUA* operon due to unfavourable interactions at the DNA-protein interface [57,58]. The resulting induction of *iscRSUA* in turn mediates induction of the *sufABCDSE* operon through binding of both apo- and holo-IscR to the type 2 motif located upstream of *sufA* [59]. The *sufABCDSE* operon is also directly regulated by OxyR and integration host factor (IHF) in response to oxidative stress [60], while the ferric uptake regulator (Fur) mediates induction of the operon in response to iron limitation [61]. A bacterial one-hybrid screen identified binding sites for seven transcriptional regulators upstream of the *M*. *tuberculosis suf* operon genes, including the redox-sensitive regulators σ^K^ and WhiB3 [62], suggesting that its expression is not solely controlled by Rv1460 (Fig 8).

Forward genetic screens predicted that *Rv1460* is not essential for the growth of *M*. *tuberculosis* in vitro [18,19]. It was therefore surprising that mutants harbouring a deletion of codons 36 to 211 of Rv1460 could not be isolated unless a second copy of the gene was present at the *attB* site in the chromosome. Subsequent attempts to remove the copy of the gene from the *attB* site of the *ΔRv1460 attB*::pMV1460 strain was 1000-fold less efficient than replacement with a functional copy of Rv1460 (Table 1), suggesting that while *Rv1460* is not essential, its loss is deleterious for *M*. *tuberculosis*. Truncation mutants in which Rv1460 was made non-functional by deletion of the DNA-binding domain and introduction of a premature stop codon at position 122, could be readily isolated. The three truncation mutants (*ΔRv1460*stop) analysed displayed a growth defect under standard culture conditions (Fig 4A–4C), although the severity of this defect was not consistent. This suggests that strains lacking functional Rv1460 may have suppressor mutations elsewhere in their chromosome.

β-galactosidase assays (Fig 2) and RT-qPCR (Fig 3) analysis confirmed that *Rv1460* functions as a repressor of its own expression, and the expression of *Rv1461*. This suggests that Rv1460 is a repressor of the *suf* operon as previous studies have demonstrated that the *Rv1460-Rv1461-Rv1462-Rv1463-csd-Rv1465-Rv1466* gene cluster is co-transcribed and co-regulated [16,52,53]. Excessive expression of the *suf* operon in *E*. *coli* was shown to inhibit growth, which correlates with the growth phenotype of the *ΔRv1460*stop mutants (Fig 4A–4C). Elevated transcription of the Fe-S cluster biogenesis machinery in the truncation mutants would cause the synthesis of Fe-S clusters to exceed the cellular demand, and in turn drive oxidative stress via the Fenton reaction [63]. Increased expression of the *suf* operon in the *ΔRv1460*stop_5.20 mutant did not result in a significant increase in the succinate dehydrogenase and aconitase activity in this strain (Fig 5). This suggests that basal expression of the operon was sufficient to meet the Fe-S cluster demand for these two enzymes.

Despite culturing the wild-type, truncation mutants (*ΔRv1460*stop) and complemented strains in iron limiting media (MM) for three growth cycles, measurement of intracellular iron levels (Table F in S1 File) indicated that iron stores were not depleted. This is consistent with a recent study demonstrating the remarkable ability of *M*. *tuberculosis* to survive for extended periods under iron limiting conditions [7]. Furthermore, the level of intracellular iron was similar between the wild-type and truncation mutants, indicating that the loss of Rv1460 did not affect *M*. *tuberculosis*’s ability to scavenge and store iron. Unexpectedly, the growth defect observed for the truncation mutants in 7H9 OADC was absent when the strains were grown in MM media (Fig 6). Transcriptomic analysis of H37Rv cultured in MM revealed that the expression of more than 150 genes were changed in response to iron limitation [54], including the down-regulation of several genes involved in energy metabolism and amino acid biosynthesis. The resulting changes in bacterial metabolism may therefore alter the growth phenotype of the mutants in MM relative to 7H9 OADC. The adaptation of *M*. *tuberculosis* to iron starvation is characterized by an induction of Fe-S cluster biogenesis and certain essential Fe-S cluster containing proteins. Since iron starvation appears to be a trigger of non-replicating persistence during infection, an understanding of Fe-S cluster biogenesis and its regulation is important for understanding the pathogenicity of *M*. *tuberculosis*.

Fe-S cluster reconstitution of Rv1460 using IscS_*E*.*coli*_ and IscU_*E*.*coli*_ suggested the formation of 2Fe-2S clusters, however since the near-UV CD spectrum for the control reaction (without Rv1460) had similar features, it was not clear if these clusters were forming on Rv1460. When the reconstitution reaction was performed with NifS, an increase in UV-visible absorbance was observed around 420 nm in the presence and absence of Rv1460, while the near-UV spectrum characteristic of 2Fe-2S clusters was only observed for the reaction containing Rv1460. This suggests that the increase in UV-visible absorbance in the NifS control reaction (without Rv1460) is from species that have a negligible near-UV CD signal [56], and that the 2Fe-2S clusters are forming on Rv1460. The peaks observed in the near-UV CD spectrum at 330 nm and 420 nm for the IscS_*E*.*coli*_ /IscU_*E*.*coli*_ control reaction may therefore be due to the formation of 2Fe-2S clusters on the scaffold IscU _*E*.*coli*_. Consistent with this hypothesis is the observation that no increase in absorbance was observed when IscU was omitted from the reaction (data not shown). Chemical reconstitution using lithium sulphide and iron, followed by buffer exchange to remove unbound species from Rv1460, revealed peaks at 330 nm and 420 nm in the near-UV spectrum, characteristic of a 2Fe-2S cluster. A control reaction lacking Rv1460 showed no peaks in this range. Taken together, these results confirm that Rv1460 is able to co-ordinate a 2Fe-2S cluster in vitro. Recombinant SufR from Synechocystis was shown to bind a 4Fe-4S cluster by electron paramagnetic resonance (EPR) spectroscopy following in vitro reconstitution [35]. Since 4Fe-4S clusters have a negligible near-UV CD signal relative to 2Fe-2S clusters, we cannot rule out that Rv1460 may also bind a 4Fe-4S cluster in vitro. Furthermore, the form of the cluster that is bound to Rv1460 in vivo remains to be confirmed. The apo- and holo-forms of Rv1460 had similar thermal denaturation profiles (Figure J, part B in S1 File), suggesting that binding of the Fe-S cluster is not required for protein stability.

Three cysteine residues, namely C203, C216 and C244, were predicted to provide ligands that coordinate the Fe-S cluster in Rv1460 [35]. We observed that protein variants harbouring serine residues at these positions replaced pMV1460 at the *attB* site in the *ΔRv1460 attB*::pMV1460 strain with the same efficiency as the empty vector (Table 1), indicating the importance of these cysteine residues in Rv1460’s function. In contrast, changing the cysteine residue not predicted to be involved in Fe-S cluster coordination (C242) had no deleterious effect on Rv1460’s function (Table 1). This suggests that the coordination of the Fe-S cluster may be affected in the cysteine variants, which in turn alters its function in *M*. *tuberculosis*. In Synechocystis the DNA binding affinity of SufR is dependent on the presence and oxidation state of the Fe-S cluster, with the holo-, oxidised form binding DNA with the highest affinity [35]. In the reporter assay in *M*. *smegmatis* all the cysteine variants demonstrated equivalent abilities to repress expression from the *Rv1460* promoter (Fig 2C). This suggests that either the altered cluster co-ordination in the variants does not change the affinity for the *Rv1460* promoter region, or that the β-galactosidase assays are not sensitive enough to report small differences in the affinity. Intrinsic differences in redox homeostasis in *M*. *smegmatis* and *M*. *tuberculosis* have been reported, and this may also impact on the functioning of the Rv1460 variants in these two organisms [64]. Alternatively, the inability of the cysteine variants to complement the *ΔRv1460* mutant may be due to altered binding and dysregulation at other loci, rather than at the *suf* operon. CHiP-seq identified binding sites for Rv1460 upstream of four other genes [52], namely: *Rv0456A*, which forms part of the *mazEF* toxin-antitoxin system; a hypothetical protein of unknown function, *Rv1116*; *Rv2107* (PE22), and *Rv3597c*, which encodes the nucleoid associated protein Lsr2 [21], supporting its role as a global regulator. Lsr2, a histone-like nucleoid structuring protein, is itself involved in regulation of numerous genes [65]. The role of Rv1460 in the regulation of these genes however remains to be elucidated, as overexpression of Rv1460 in *M*. *tuberculosis* did not significantly alter their mRNA expression levels [66].

## Conclusions

In this study, we demonstrate that the SufR homologue Rv1460 is a transcriptional repressor of the *suf* operon in *M*. *tuberculosis*. Loss of Rv1460 is deleterious for growth of *M*. *tuberculosis* under standard culture conditions, while growth in iron limiting media is not affected. Furthermore, we show that Rv1460 binds an Fe-S cluster, and demonstrate that three cysteine residues predicted to coordinate the Fe-S cluster are important for the functioning of this regulator in *M*. *tuberculosis*.

## Supporting information

S1 File Supporting information. (DOCX)

## References

[1] WHO. Global tuberculosis report. 20^th^ Edition. Geneva: WHO 2015 Available from: http://www.who.int/tb/publications/global_report/en/. (Accessed March 2. 2016).

[2] Schnappinger D, Ehrt S, Voskuil MI, Liu Y, Mangan JA, Monahan IM, et al Transcriptional adaptation of *Mycobacterium tuberculosis* within macrophages: Insights into the phagosomal environment. J Exp Med. 2003 9 1;198(5):693–704. doi: 10.1084/jem.20030846 12953091 10.1084/jem.20030846 PMC2194186

[3] Aly S, Wagner K, Keller C, Malm S, Malzan A, Brandau S, et al Oxygen status of lung granulomas in *Mycobacterium tuberculosis*‐infected mice. J Pathol. 2006 11 1;210(3):298–305. doi: 10.1002/path.2055 17001607 10.1002/path.2055

[4] Via LE, Lin PL, Ray SM, Carrillo J, Allen SS, Eum SY, et al Tuberculous granulomas are hypoxic in guinea pigs, rabbits, and nonhuman primates. Infect Immun. 2008 6;76(6):2333–40. doi: 10.1128/IAI.01515-07 18347040 10.1128/IAI.01515-07 PMC2423064

[5] Wayne LG. In vitro model of hypoxically induced nonreplicating persistence of *Mycobacterium tuberculosis*. Methods Mol Med. 2001;54:247–69. doi: 10.1385/1-59259-147-7:247 21341080 10.1385/1-59259-147-7:247

[6] Wayne LG, Hayes LG. Nitrate reduction as a marker for hypoxic shiftdown of *Mycobacterium tuberculosis*. Tuber Lung Dis Off J Int Union Tuberc Lung Dis. 1998;79(2):127–32. 10.1054/tuld.1998.0015 10645451

[7] Kurthkoti K, Amin H, Marakalala MJ, Ghanny S, Subbian S, Sakatos A, et al The capacity of *Mycobacterium tuberculosis* to survive iron starvation might enable it to persist in iron-deprived microenvironments of human granulomas. mBio. 2017 8 15;8(4). 10.1128/mBio.01092-17 PMC5559634 28811344

[8] Fontecave M. Iron-sulfur clusters: ever-expanding roles. Nat Chem Biol. 2006 4;2(4):171–4. doi: 10.1038/nchembio0406-171 16547473 10.1038/nchembio0406-171

[9] Jacobson MR, Cash VL, Weiss MC, Laird NF, Newton WE, Dean DR. Biochemical and genetic analysis of the *nifUSVWZM* cluster from *Azotobacter vinelandii*. Mol Gen Genet MGG. 1989 10;219(1–2):49–57. 2615765 10.1007/BF00261156

[10] Zheng L, Cash VL, Flint DH, Dean DR. Assembly of iron-sulfur clusters identification of an *iscSUA-hscBA-fdx* gene cluster from *Azotobacter vinelandii*. J Biol Chem. 1998 5 22;273(21):13264–72. 9582371 10.1074/jbc.273.21.13264

[11] Takahashi Y, Tokumoto U. A third bacterial system for the assembly of iron-sulfur clusters with homologs in archaea and plastids. J Biol Chem. 2002 8 9;277(32):28380–3. doi: 10.1074/jbc.C200365200 12089140 10.1074/jbc.C200365200

[12] Schwartz CJ, Djaman O, Imlay JA, Kiley PJ. The cysteine desulfurase, IscS, has a major role in in vivo Fe-S cluster formation in *Escherichia coli*. Proc Natl Acad Sci U S A. 2000 8 1;97(16):9009–14. doi: 10.1073/pnas.160261497 10908675 10.1073/pnas.160261497 PMC16812

[13] Nachin L, Loiseau L, Expert D, Barras F. SufC: An unorthodox cytoplasmic ABC/ATPase required for [Fe-S] biogenesis under oxidative stress. EMBO J. 2003 2 3;22(3):427–37. doi: 10.1093/emboj/cdg061 12554644 10.1093/emboj/cdg061 PMC140745

[14] Dai Y, Outten FW. The *E*. *coli* SufS-SufE sulfur transfer system is more resistant to oxidative stress than IscS-IscU. FEBS Lett. 2012 11 16;586(22):4016–22. doi: 10.1016/j.febslet.2012.10.001 23068614 10.1016/j.febslet.2012.10.001 PMC3511050

[15] Outten FW, Djaman O, Storz G. A *suf* operon requirement for Fe-S cluster assembly during iron starvation in *Escherichia coli*. Mol Microbiol. 2004 5;52(3):861–72. doi: 10.1111/j.1365-2958.2004.04025.x 15101990 10.1111/j.1365-2958.2004.04025.x

[16] Huet G, Daffé M, Saves I. Identification of the *Mycobacterium tuberculosis* SUF machinery as the exclusive mycobacterial system of [Fe-S] cluster assembly: evidence for its implication in the pathogen’s survival. J Bacteriol. 2005 9;187(17):6137–46. doi: 10.1128/JB.187.17.6137-6146.2005 16109955 10.1128/JB.187.17.6137-6146.2005 PMC1196142

[17] Huet G, Daffé M, Saves I. Identification of the *Mycobacterium tuberculosis* SUF machinery as the exclusive mycobacterial system of [Fe-S] cluster assembly: evidence for its implication in the pathogen’s survival. J Bacteriol. 2005 9;187(17):6137–46. doi: 10.1128/JB.187.17.6137-6146.2005 16109955 10.1128/JB.187.17.6137-6146.2005 PMC1196142

[18] Sassetti CM, Boyd DH, Rubin EJ. Genes required for mycobacterial growth defined by high density mutagenesis. Mol Microbiol. 2003 4;48(1):77–84. 12657046 10.1046/j.1365-2958.2003.03425.x

[19] Griffin JE, Gawronski JD, Dejesus MA, Ioerger TR, Akerley BJ, Sassetti CM. High-resolution phenotypic profiling defines genes essential for mycobacterial growth and cholesterol catabolism. PLoS Pathog. 2011 9;7(9):e1002251 doi: 10.1371/journal.ppat.1002251 21980284 10.1371/journal.ppat.1002251 PMC3182942

[20] Rybniker J, Pojer F, Marienhagen J, Kolly GS, Chen JM, van Gumpel E, et al The cysteine desulfurase IscS of *Mycobacterium tuberculosis* is involved in iron-sulfur cluster biogenesis and oxidative stress defence. Biochem J. 2014 5 1;459(3):467–78. doi: 10.1042/BJ20130732 24548275 10.1042/BJ20130732

[21] Cole ST, Brosch R, Parkhill J, Garnier T, Churcher C, Harris D, et al Deciphering the biology of *Mycobacterium tuberculosis* from the complete genome sequence. Nature. 1998 6 11;393(6685):537–44. doi: 10.1038/31159 9634230 10.1038/31159

[22] Song H, Hu W, Naowarojna N, Sae Her A, Wang S, Desai R, Qin L, Chen X, Liu P. Mechanistic studies of a novel C-S lyase in ergothioneine biosynthesis: the involvement of a sulfenic acid intermediate. Scientific Reports 5 2015 doi: 10.1038/srep11870 26149121 10.1038/srep11870 PMC4493562

[23] Hirabayashi K, Yuda E, Tanaka N, Katayama S, Iwasaki K, Matsumoto T, et al Functional dynamics revealed by the structure of the SufBCD complex, a novel ATP-binding cassette (ABC) protein that serves as a scaffold for iron-sulfur cluster biogenesis. J Biol Chem. 2015 12 11;290(50):29717–31. doi: 10.1074/jbc.M115.680934 26472926 10.1074/jbc.M115.680934 PMC4705970

[24] Outten FW, Wood MJ, Munoz FM, Storz G. The SufE protein and the SufBCD complex enhance SufS cysteine desulfurase activity as part of a sulfur transfer pathway for Fe-S cluster assembly in *Escherichia coli*. J Biol Chem. 2003 11 14;278(46):45713–9. doi: 10.1074/jbc.M308004200 12941942 10.1074/jbc.M308004200

[25] Tian T, He H, Liu X-Q. The SufBCD protein complex is the scaffold for iron-sulfur cluster assembly in *Thermus thermophiles* HB8. Biochem Biophys Res Commun. 2014 1 10;443(2):376–81. doi: 10.1016/j.bbrc.2013.11.131 24333431 10.1016/j.bbrc.2013.11.131

[26] Wollers S, Layer G, Garcia-Serres R, Signor L, Clemancey M, Latour J-M, et al Iron-sulfur (Fe-S) cluster assembly: The SufBCD complex is a new type of Fe-S scaffold with a flavin redox cofactor. J Biol Chem. 2010 7 23;285(30):23331–41. doi: 10.1074/jbc.M110.127449 20460376 10.1074/jbc.M110.127449 PMC2906325

[27] Saini A, Mapolelo DT, Chahal HK, Johnson MK, Outten FW. SufD and SufC ATPase activity are required for iron acquisition during in vivo Fe-S cluster formation on SufB. Biochemistry (Mosc). 2010 11 2;49(43):9402–12. 10.1021/bi1011546 PMC3004146 20857974

[28] Huet G, Castaing J-P, Fournier D, Daffé M, Saves I. Protein splicing of SufB is crucial for the functionality of the *Mycobacterium tuberculosis* SUF Machinery. J Bacteriol. 2006 5;188(9):3412–4. doi: 10.1128/JB.188.9.3412-3414.2006 16621837 10.1128/JB.188.9.3412-3414.2006 PMC1447444

[29] Saves I, Lewis L-A, Westrelin F, Warren R, Daffé M, Masson J-M. Specificities and functions of the *recA* and *pps1* intein genes of *Mycobacterium tuberculosis* and application for diagnosis of tuberculosis. J Clin Microbiol. 2002 3;40(3):943–50. doi: 10.1128/JCM.40.3.943-950.2002 11880421 10.1128/JCM.40.3.943-950.2002 PMC120251

[30] Topilina NI, Green CM, Jayachandran P, Kelley DS, Stanger MJ, Piazza CL, et al SufB intein of *Mycobacterium tuberculosis* as a sensor for oxidative and nitrosative stresses. Proc Natl Acad Sci U S A. 2015 8 18;112(33):10348–53. doi: 10.1073/pnas.1512777112 26240361 10.1073/pnas.1512777112 PMC4547236

[31] Voskuil MI, Bartek IL, Visconti K, Schoolnik GK. The response of *Mycobacterium tuberculosis* to reactive oxygen and nitrogen species. Front Microbiol. 2011;2:105 doi: 10.3389/fmicb.2011.00105 21734908 10.3389/fmicb.2011.00105 PMC3119406

[32] Betts JC, Lukey PT, Robb LC, McAdam RA, Duncan K. Evaluation of a nutrient starvation model of *Mycobacterium tuberculosis* persistence by gene and protein expression profiling. Mol Microbiol. 2002 2;43(3):717–31. 11929527 10.1046/j.1365-2958.2002.02779.x

[33] Kumar M, Khan FG, Sharma S, Kumar R, Faujdar J, Sharma R, et al Identification of *Mycobacterium tuberculosis* genes preferentially expressed during human infection. Microb Pathog. 2011 1;50(1):31–8. doi: 10.1016/j.micpath.2010.10.003 21035536 10.1016/j.micpath.2010.10.003

[34] Wang T, Shen G, Balasubramanian R, McIntosh L, Bryant DA, Golbeck JH. The *sufR* gene (*sll0088* in *Synechocystis* sp. strain PCC 6803) functions as a repressor of the *sufBCDS* operon in iron-sulfur cluster biogenesis in cyanobacteria. J Bacteriol. 2004 2;186(4):956–67. doi: 10.1128/JB.186.4.956-967.2004 14761990 10.1128/JB.186.4.956-967.2004 PMC344230

[35] Shen G, Balasubramanian R, Wang T, Wu Y, Hoffart LM, Krebs C, et al SufR coordinates two [4Fe-4S]^2+, 1+^ clusters and functions as a transcriptional repressor of the *sufBCDS* operon and an autoregulator of *sufR* in Cyanobacteria. J Biol Chem. 2007 11 2;282(44):31909–19. doi: 10.1074/jbc.M705554200 17827500 10.1074/jbc.M705554200

[36] Gold B, Rodriguez GM, Marras SA, Pentecost M, Smith I. The *Mycobacterium tuberculosis* IdeR is a dual functional regulator that controls transcription of genes involved in iron acquisition, iron storage and survival in macrophages. Mol Microbiol. 2001 11;42(3):851–65. 11722747 10.1046/j.1365-2958.2001.02684.x

[37] Pandey R, Rodriguez GM. IdeR is required for iron homeostasis and virulence in *Mycobacterium tuberculosis*. Mol Microbiol. 2014 1;91(1):98–109. doi: 10.1111/mmi.12441 24205844 10.1111/mmi.12441 PMC3902104

[38] Wang C., Lee J., Deng Y., Tao F. and Zhang L.-H. ARF-TSS: an alternative method for identification of transcription start site in bacteria. BioTechniques. 2012;52 doi: 10.2144/000113858 26307248 10.2144/000113858

[39] Edelheit O, Hanukoglu A, Hanukoglu I. Simple and efficient site-directed mutagenesis using two single-primer reactions in parallel to generate mutants for protein structure-function studies. BMC Biotechnol. 2009 6 30;9:61–8. doi: 10.1186/1472-6750-9-61 19566935 10.1186/1472-6750-9-61 PMC2711942

[40] Parish T, Stoker NG. Use of a flexible cassette method to generate a double unmarked *Mycobacterium tuberculosis tlyA plcABC* mutant by gene replacement. Microbiol Read Engl. 2000 8;146:1969–75. 10.1099/00221287-146-8-1969 10931901

[41] Springer B, Sander P, Sedlacek L, Ellrott K, Böttger EC. Instability and site-specific excision of integration-proficient mycobacteriophage L5 plasmids: development of stably maintained integrative vectors. Int J Med Microbiol. 2001 3;290(8):669–75. doi: 10.1016/S1438-4221(01)80004-7 11310445 10.1016/S1438-4221(01)80004-7

[42] Tian J, Bryk R, Itoh M, Suematsu M, Nathan C. Variant tricarboxylic acid cycle in *Mycobacterium tuberculosis*: identification of alpha-ketoglutarate decarboxylase. Proc Natl Acad Sci USA. 2005 7 26;102(30):10670–5. doi: 10.1073/pnas.0501605102 16027371 10.1073/pnas.0501605102 PMC1180764

[43] Munujos P, Coll-Cantí J, González-Sastre F, Gella FJ. Assay of succinate dehydrogenase activity by a colorimetric-continuous method using iodonitrotetrazolium chloride as electron acceptor. Anal Biochem. 1993 8 1;212(2):506–9. 8214593 10.1006/abio.1993.1360

[44] Riemer J, Hoepken HH, Czerwinska H, Robinson SR, Dringen R. Colorimetric ferrozine-based assay for the quantitation of iron in cultured cells. Anal Biochem. 2004 8 15;331(2):370–5. doi: 10.1016/j.ab.2004.03.049 15265744 10.1016/j.ab.2004.03.049

[45] Prischi F, Pastore C, Carroni M, Iannuzzi C, Adinolfi S, Temussi P, et al Of the vulnerability of orphan complex proteins: The case study of the *E*. *coli* IscU and IscS proteins. Protein Expr Purif. 2010 10;73(2):161–6. doi: 10.1016/j.pep.2010.05.003 20471481 10.1016/j.pep.2010.05.003

[46] Tropea JE, Cherry S, Waugh DS. Expression and purification of soluble His(6)-tagged TEV protease. Methods Mol Biol Clifton NJ. 2009;498:297–307. 10.1007/978-1-59745-196-3_19 18988033

[47] Adinolfi S, Iannuzzi C, Prischi F, Pastore C, Iametti S, Martin SR, et al Bacterial frataxin CyaY is the gatekeeper of iron-sulfur cluster formation catalyzed by IscS. Nat Struct Mol Biol. 2009 4;16(4):390–6. doi: 10.1038/nsmb.1579 19305405 10.1038/nsmb.1579

[48] Adrover M, Howes BD, Iannuzzi C, Smulevich G, Pastore A. Anatomy of an iron-sulfur cluster scaffold protein: Understanding the determinants of [2Fe-2S] cluster stability on IscU. Biochim Biophys Acta. 2015 6;1853(6):1448–56. doi: 10.1016/j.bbamcr.2014.10.023 25447544 10.1016/j.bbamcr.2014.10.023

[49] Smith LJ, Stapleton MR, Fullstone GJM, Crack JC, Thomson AJ, Le Brun NE, et al *Mycobacterium tuberculosis* WhiB1 is an essential DNA-binding protein with a nitric oxide-sensitive iron-sulfur cluster. Biochem J. 2010 12 15;432(3):417–27. doi: 10.1042/BJ20101440 20929442 10.1042/BJ20101440 PMC2992795

[50] Boyd JM, Sondelski JL, Downs DM. Bacterial ApbC protein has two biochemical activities that are required for in vivo function. J Biol Chem. 2009 1 2;284(1):110–8. doi: 10.1074/jbc.M807003200 19001370 10.1074/jbc.M807003200 PMC2610507

[51] Cortes T, Schubert OT, Rose G, Arnvig KB, Comas I, Aebersold R, et al Genome-wide mapping of transcriptional start sites defines an extensive leaderless transcriptome in *Mycobacterium tuberculosis*. Cell Rep. 2013 11 27;5(4):1121–31. doi: 10.1016/j.celrep.2013.10.031 24268774 10.1016/j.celrep.2013.10.031 PMC3898074

[52] Minch KJ, Rustad TR, Peterson EJR, Winkler J, Reiss DJ, Ma S, et al The DNA-binding network of *Mycobacterium tuberculosis*. Nat Commun. 2015;6:5829 doi: 10.1038/ncomms6829 25581030 10.1038/ncomms6829 PMC4301838

[53] Roback P, Beard J, Baumann D, Gille C, Henry K, Krohn S, et al A predicted operon map for *Mycobacterium tuberculosis*. Nucleic Acids Res. 2007 8;35(15):5085–95. doi: 10.1093/nar/gkm518 17652327 10.1093/nar/gkm518 PMC1976454

[54] Rodriguez GM, Voskuil MI, Gold B, Schoolnik GK, Smith I. *ideR*, An essential gene in *Mycobacterium tuberculosis*: role of IdeR in iron-dependent gene expression, iron metabolism, and oxidative stress response. Infect Immun. 2002 7;70(7):3371–81. doi: 10.1128/IAI.70.7.3371-3381.2002 12065475 10.1128/IAI.70.7.3371-3381.2002 PMC128082

[55] Yan R, Adinolfi S, Pastore A. Ferredoxin, in conjunction with NADPH and ferredoxin-NADP reductase, transfers electrons to the IscS/IscU complex to promote iron-sulfur cluster assembly. Biochim Biophys Acta. 2015 9;1854(9):1113–7. doi: 10.1016/j.bbapap.2015.02.002 25688831 10.1016/j.bbapap.2015.02.002 PMC4547094

[56] Fox NG, Das D, Chakrabarti M, Lindahl PA, Barondeau DP. Frataxin Accelerates [2Fe-2S] Cluster Formation on the Human Fe-S Assembly Complex. Biochemistry (Mosc). 2015 6 30;54(25):3880–9. 10.1021/bi5014497 PMC4675465 26016518

[57] Rajagopalan S, Teter SJ, Zwart PH, Brennan RG, Phillips KJ, Kiley PJ. Studies of IscR reveal a unique mechanism for metal-dependent regulation of DNA binding specificity. Nat Struct Mol Biol. 2013 6;20(6):740–7. doi: 10.1038/nsmb.2568 23644595 10.1038/nsmb.2568 PMC3676455

[58] Santos JA, Alonso-García N, Macedo-Ribeiro S, Pereira PJB. The unique regulation of iron-sulfur cluster biogenesis in a Gram-positive bacterium. Proc Natl Acad Sci U S A. 2014 6 3;111(22):2251–60. 10.1073/pnas.1322728111 PMC4050560 24847070

[59] Mettert EL, Kiley PJ. Coordinate regulation of the suf and isc Fe-S cluster biogenesis pathways by IscR is essential for viability of *Escherichia coli*. J Bacteriol. 2014 12;196(24):4315–23. doi: 10.1128/JB.01975-14 25266384 10.1128/JB.01975-14 PMC4248859

[60] Lee J-H, Yeo W-S, Roe J-H. Induction of the *sufA* operon encoding Fe-S assembly proteins by superoxide generators and hydrogen peroxide: involvement of OxyR, IHF and an unidentified oxidant-responsive factor. Mol Microbiol. 2004 3 1;51(6):1745–55. 15009899 10.1111/j.1365-2958.2003.03946.x

[61] Lee J. H., Yeo W. S. and Roe J. H. Regulation of the *sufABCESE* operon by FurA. J. Microbiol. Seoul National University, Seoul, Republic of Korea. 2003 Available from: http://agris.fao.org/agris-search/search.do?recordID = KR2004005614 (Accessed May 16, 2016).

[62] Guo M, Feng H, Zhang J, Wang W, Wang Y, Li Y, et al Dissecting transcription regulatory pathways through a new bacterial one-hybrid reporter system. Genome Res. 2009 7;19(7):1301–8. doi: 10.1101/gr.086595.108 19228590 10.1101/gr.086595.108 PMC2704442

[63] Imlay JA. Iron-sulphur clusters and the problem with oxygen. Mol Microbiol. 2006 2;59(4):1073–82. doi: 10.1111/j.1365-2958.2006.05028.x 16430685 10.1111/j.1365-2958.2006.05028.x

[64] Tyagi P, Dharmaraja AT, Bhaskar A, Chakrapani H, Singh A. *Mycobacterium tuberculosis* has diminished capacity to counteract redox stress induced by elevated levels of endogenous superoxide. Free Radic Biol Med. 2015 7;84:344–54. doi: 10.1016/j.freeradbiomed.2015.03.008 25819161 10.1016/j.freeradbiomed.2015.03.008 PMC4459714

[65] Colangeli R, Helb D, Vilchèze C, Hazbón MH, Lee C-G, Safi H, et al Transcriptional regulation of multi-drug tolerance and antibiotic-induced responses by the histone-like protein Lsr2 in *M*. *tuberculosis*. PLoS Pathog. 2007 6;3(6):e87 doi: 10.1371/journal.ppat.0030087 17590082 10.1371/journal.ppat.0030087 PMC1894825

[66] Rustad TR, Minch KJ, Ma S, Winkler JK, Hobbs S, Hickey M, et al Mapping and manipulating the *Mycobacterium tuberculosis* transcriptome using a transcription factor overexpression-derived regulatory network. Genome Biol. 2014 11 3;15(11):502 doi: 10.1186/s13059-014-0502-3 25380655 10.1186/s13059-014-0502-3 PMC4249609

