# Correction: Rv1460, a SufR homologue, is a repressor of the *suf* operon in *Mycobacterium tuberculosis*

**DOI:** 10.1371/journal.pone.0208568

**Published:** 2018-11-29

**Authors:** The PLOS ONE Staff

Figs 1, 2, 6, 7, and 8 are incorrect. The figure captions appear in the correct order. The publisher apologizes for the errors. Please view the corrected versions of Figs 1, 2, 6, 7 and 8 here.

**Fig 1:**
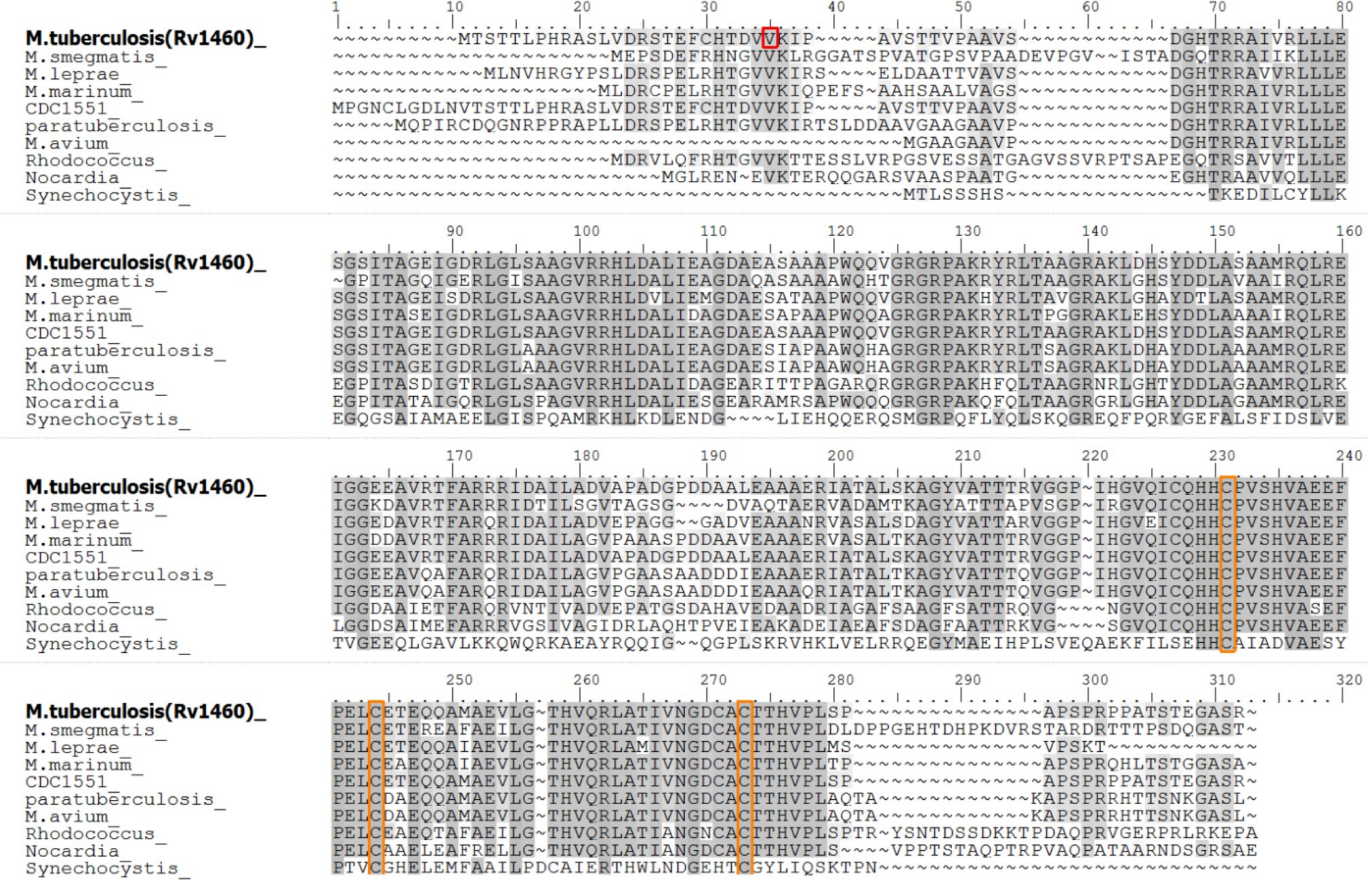
Rv1460 is a SufR homologue. Maestro Multiple Sequence Viewer multiple alignment of Rv1460 [ACJ83238] with homologues in selected mycobacteria and SufR homologues present in other organisms. The re-annotated start site of Rv1460 is indicated by the red box. Conserved cysteine residues are indicated by the orange boxes. *Mycobacterium smegmatis*, transcriptional regulator [WP_011728830]; *Mycobacterium leprae*, transcriptional regulator [WP_010907825]; *Mycobacterium marinum* E11, transcriptional regulatory protein [CDM76334]; *Mycobacterium tuberculosis* CDC1551, conserved hypothetical protein [AAK45771]; *Mycobacterium avium* subsp. paratuberculosis K-10, hypothetical protein MAP_1186 [AAS03503]; *Mycobacterium avium* 104, DNA-binding protein [ABK68668]; *Rhodococcus fascians*, transcriptional regulator [WP_037190040]; *Nocardia veteran*, transcriptional regulator [WP_051031599] and *Synechocystis* sp. PCC 6803, SufR [WP 020862050].

**Fig 2:**
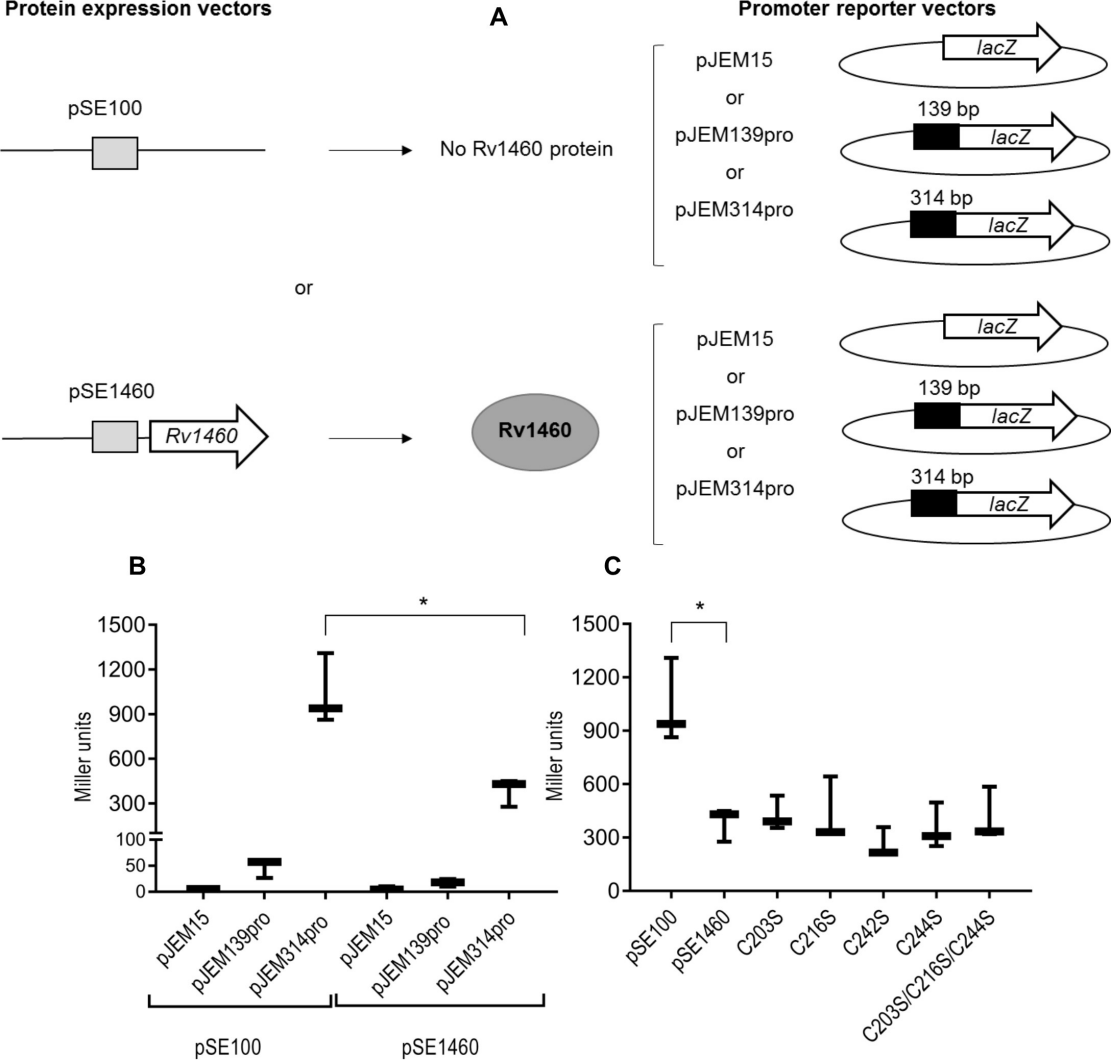
Rv1460 represses its own expression. (A) Schematic representation of the β-galactosidase assay procedure. Each reporter vector (pJEM15, pJEM139pro or pJEM314pro) (containing the 139 bp or 314 bp regions upstream of *Rv1460* fused to a *lacZ* reporter gene) was co-transformed into *M*. *smegmatis* with a protein expression vector either encoding no protein (pSE100) or encoding Rv1460 (indicated by the grey oval). (B) β-galactosidase activity from the 139 bp and 314 bp promoter fragments with (pSE1460) and without (pSE100) co-expression of Rv1460 in *M*. *smegmatis*. Transcriptional repression by wild-type or variants of Rv1460 results in a decrease in β-galactosidase activity, which is expressed in Miller units calculated as follows: 200 × (change in OD_450nm_) per mg protein per min. The results shown are the mean and standard deviation for three experiments. Statistical analysis compared the mean activity for each plasmid with or without co-expression of Rv1460 e.g. pJEM15 (pSE100) vs. pJEM15 (pSE1460) using an unpaired t-test (*p ≤0.05). (C) β-galactosidase activity from the 314 bp promoter (pJEM314pro) when wild-type (pSE1460) or mutated forms of Rv1460 (pSE_C203S or pSE_C216S or pSE_C242S or pSE_C244S or pSE_C203S/C216S/C244S) are expressed in *M*. *smegmatis*. The results shown are the mean and standard deviation for three experiments. Statistical analysis compared the mean activity for wild-type with each variant using an unpaired t-test.

**Fig 6:**
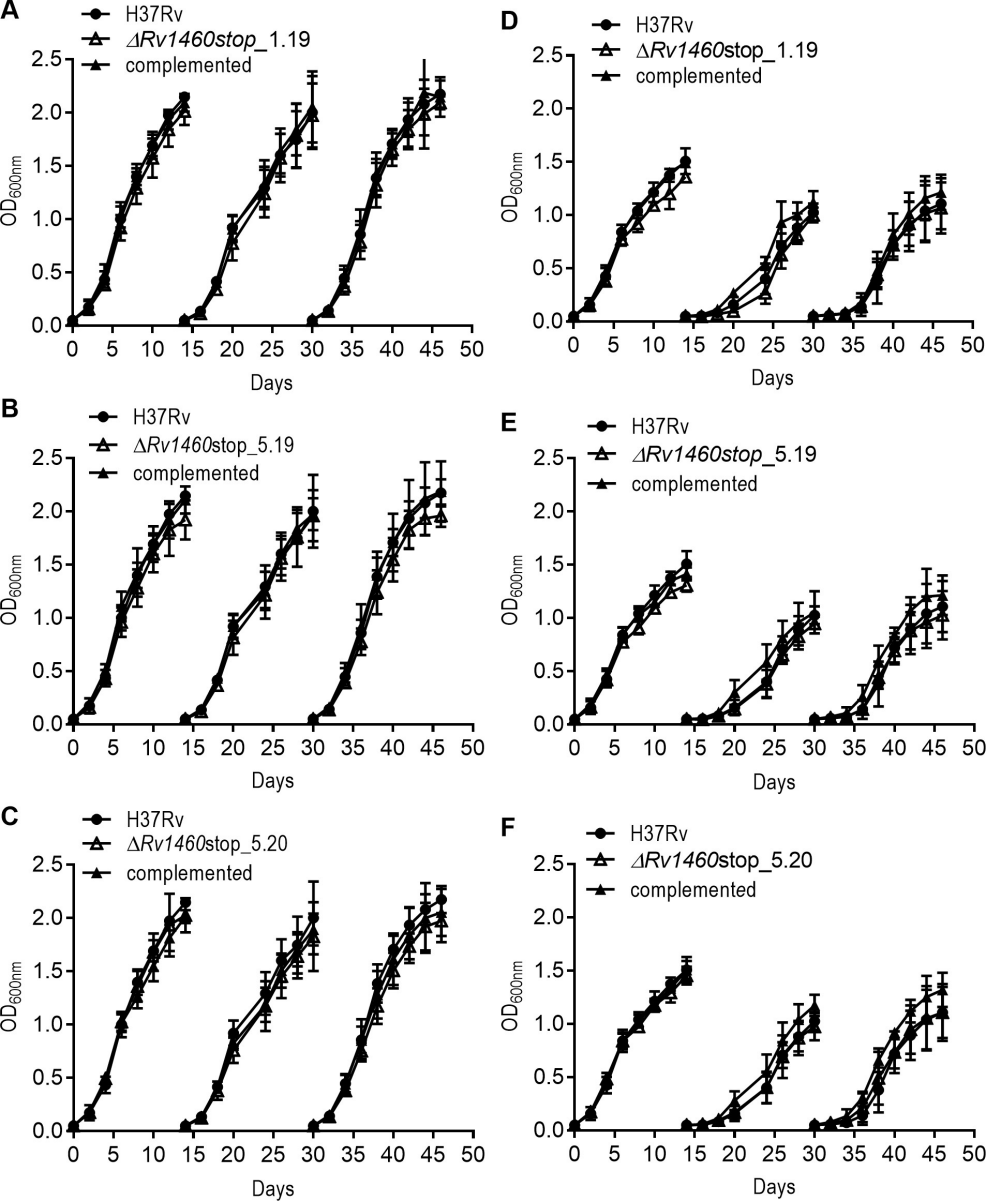
Rv1460 truncation mutants are not impaired for growth under iron-limiting conditions. Growth of H37Rv (wild-type), three truncation mutants (Δ*Rv1460*stop) and complemented strains in (A–C) MM + Fe^+3^ and (D–F) MM for three growth cycles (subcultured on day 14 to an OD_600nm_ of 0.05). The results shown are the mean and standard deviation of three experiments.

**Fig 7:**
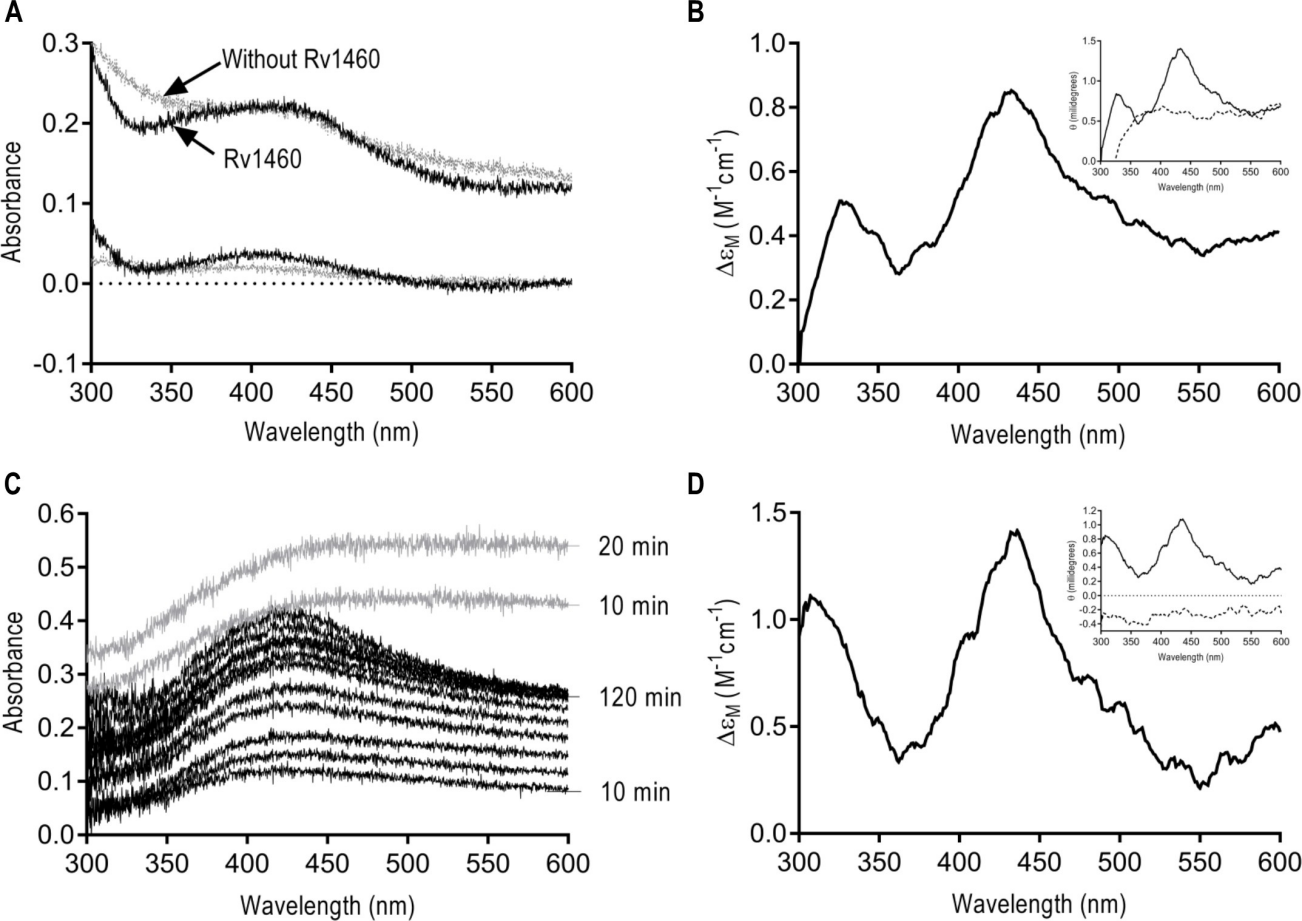
Rv1460 binds an Fe-S cluster. (A) UV-visible spectrum of NifS reconstitution reaction (at 1 and 20 hours) containing Rv1460 (50 μM) (black) or without Rv1460 (grey). The absorbance at 2 minutes was used as a blank for the reaction. (B) Near-UV CD spectrum of NifS reconstitution reaction containing Rv1460 (50 μM). Insert shows the near-UV CD spectrum (Ellipticity in millidegrees) of reactions with (solid line) and without Rv1460 (dashed line). (C) UV-visible spectrum of lithium sulphide reconstitution reaction with (black) and without Rv1460 (50 μM) over time. The absorbance at 2 minutes was used as a blank for the reaction. UV-visible spectrum of the reactions after buffer exchange are indicated in Figure K. in S1 file (D) Near-UV CD spectrum of lithium sulphide reconstitution reaction after buffer exchange containing (23 μM) Rv1460. Insert shows the near-UV CD (Ellipticity in millidegrees) of reactions with (solid line) and without Rv1460 (dashed line) after buffer exchange.

**Fig 8:**
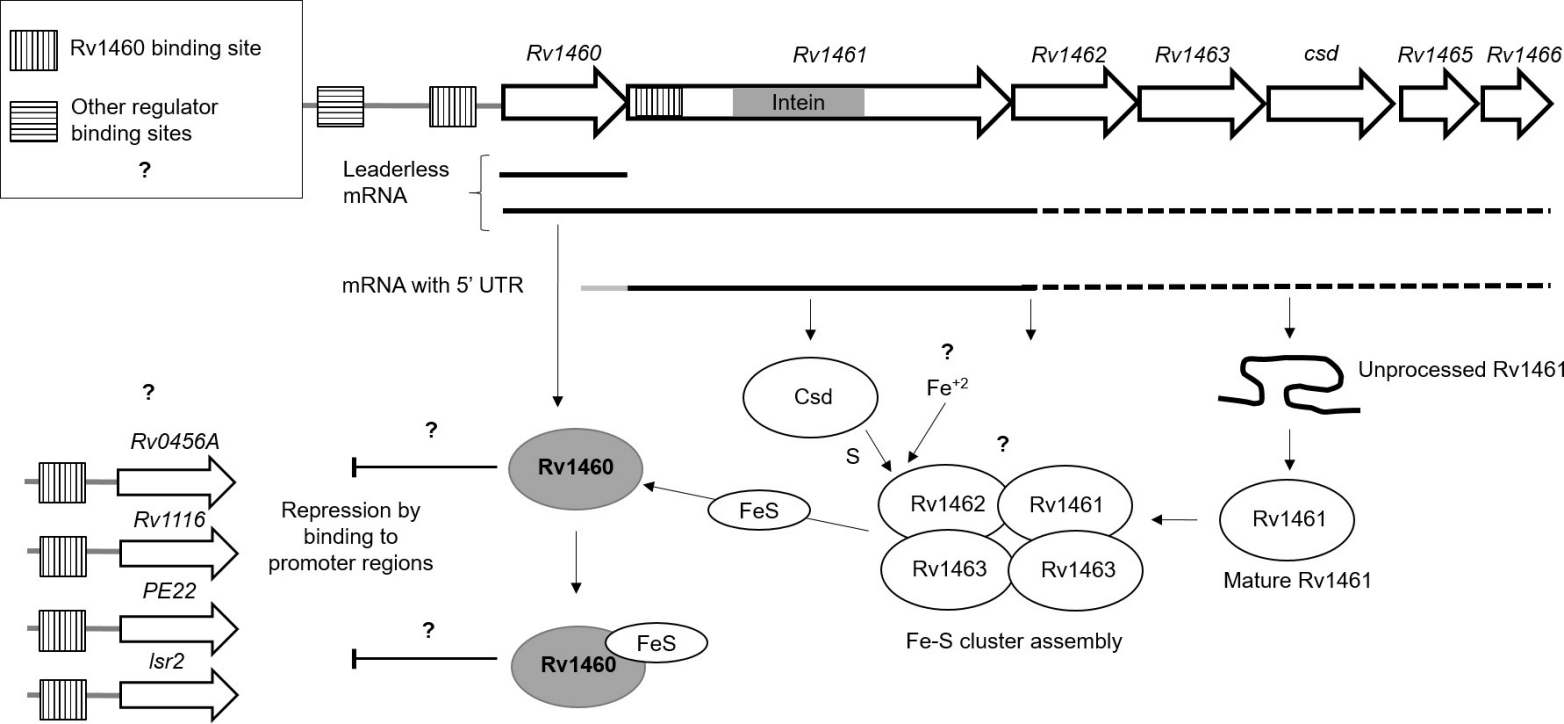
Fe-S cluster assembly is regulated by multiple mechanisms in *M*. *tuberculosis*. *Rv1460* is transcribed independently from the operon allowing differential expression and regulation by the repressor. Initiation of translation is leaderless upstream of *Rv1460*, while a 5’-UTR containing a ribosome binding site regulates translation of *Rv1461*. The intein within Rv1461 must be resolved before it is functional and may represent an additional level of regulation of the system. Rv1460 is predicted to bind within the promoter region upstream of Rv1460 as well as within Rv1461 (as indicated by the dashed line boxes). Binding sites for other regulators within the Rv1460 promoter region and within the operon are also predicted, providing another level of regulation. Binding of an Fe-S cluster may change Rv1460’s affinity for DNA. Genes are not drawn to scale. The association of the Fe-S cluster machinery is inferred from other bacterial SUF systems,but has not been experimentally validated in mycobacteria. Cysteine is provided by the cysteine desulphurase, while the source of iron is unknown. The oligomeric state of Rv1460 is not indicated since it has not been confirmed, and the ratio of Fe-S cluster to Rv1460 protein is unknown.
