# Supplementary material for: Rv1460, a SufR homologue, is a repressor of the suf operon in Mycobacterium tuberculosis
Source: PLoS One. 2018 Jul 6;13(7):e0200145. doi: 10.1371/journal.pone.0200145 (PMC6034842; doi:10.1371/journal.pone.0200145)
Supplement: S1 File — (DOCX) [file pone.0200145.s001.docx]

# S1 File. Supporting information

**Rv1460, a SufR homologue, is a repressor of the *suf* operon in *Mycobacterium tuberculosis***

Danicke Willemse^1^, Brandon Weber^2^, Laura Masino^3^, Robin M. Warren^1^, Salvatore Adinolfi^4^, Annalisa Pastore^5^, Monique J. Williams^1*^

^1^DST-NRF Centre of Excellence for Biomedical Tuberculosis Research; SAMRC Centre for Tuberculosis Research; Division of Molecular Biology and Human Genetics, Faculty of Medicine and Health Sciences, Stellenbosch University, Tygerberg, South Africa

^2^Electron Microscope Unit, University of Cape Town, Cape Town, South Africa

^3^Structural Biology Science Technology Platform, The Francis Crick Institute, 1 Midland Road, London NW1 1AT, United Kingdom

^4^ Pharmaceutical Science and Technology, University of Turin, Turin, Italy

^5^Department of Basic and Clinical Neuroscience, Maurice Wohl Institute, King's College London, London, United Kingdom

*Corresponding author

E-mail: moniquejw@sun.ac.za (MJW)

**
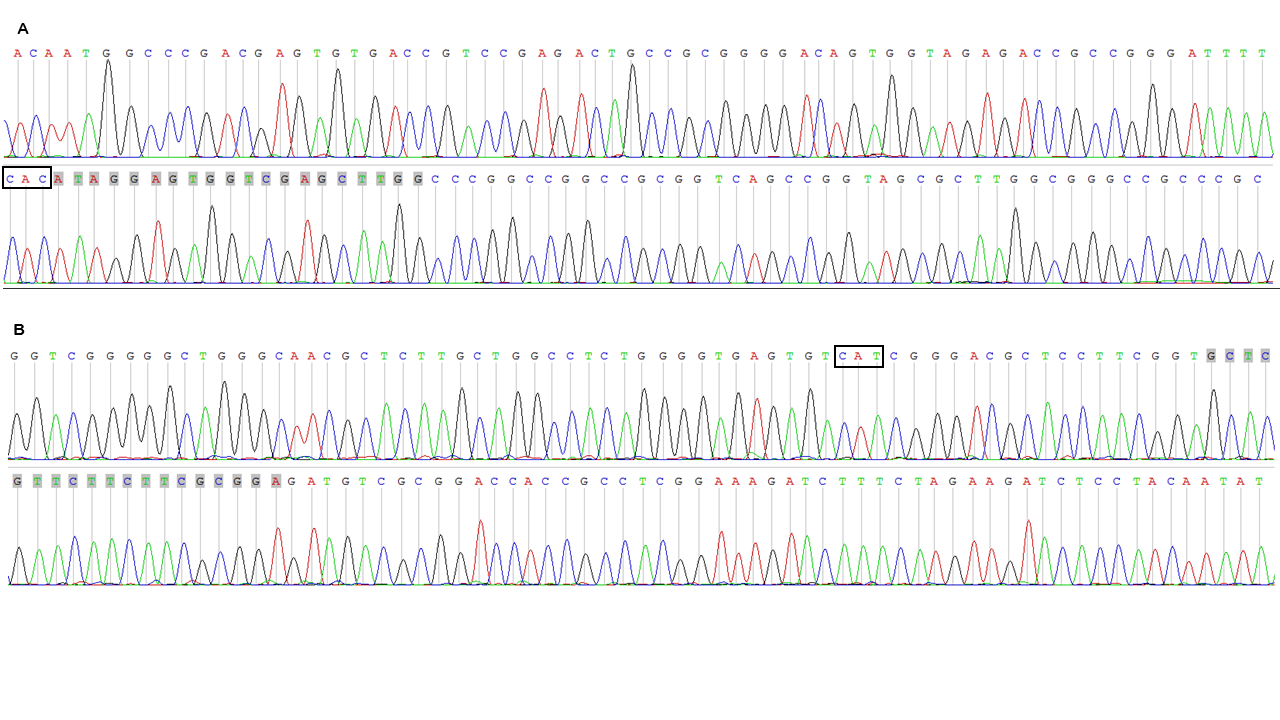
**

**II**

**I**

**
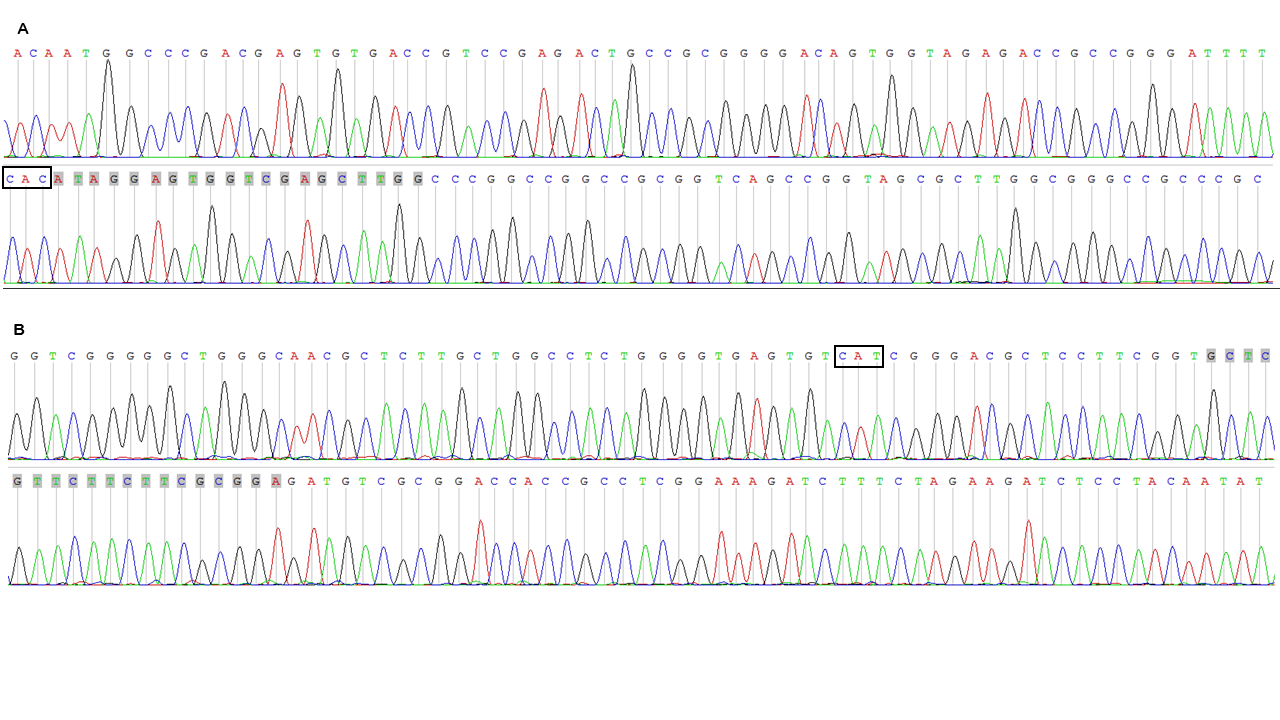
 Figure A. Sanger sequencing of clones obtained using the adaptor- and radioactivity-free identification of transcriptional start sites (ARF-TSS) method.** Grey highlighted bases indicate phosphorylated reverse primers used in the reverse transcription step. Black boxes indicate the bases of the start codon of (A) *Rv1460* and (B) *Rv1461*.

**Figure B. Genotypic characterization of the *M. tuberculosis* Δ*Rv1460* *attB*::pMV1460 strains.** The restriction maps of the (A) H37Rv (wild type) and (B) Δ*Rv1460* *attB*::pMV1460 strain. Regions of homology that were cloned into the suicide delivery vector used for allelic exchange mutagenesis are denoted by hashed boxes and position of PCR screening primers Scr1, 2 and 3 are indicated. (C) PCR screening results are 843 bp and 408 bp products for Δ*Rv1460* *attB*::pMV1460 strain and wild-type respectively. Southern blots using (D) NcoI digested chromosomal DNA and ‘*Rv1460* upstream’ region as probe and (E) ApaI digested chromosomal DNA and ‘*Rv1460* downdel’ region as probe. Two Δ*Rv1460* *attB*::pMV1460 strains were independently isolated and confirmed, but only one was used for subsequent experiments.

**Figure C. Genotypic characterization of the *M. tuberculosis* truncation (Δ*Rv1460*stop) mutants.** The restriction maps of the (A) H37Rv (wild type) and (B) Δ*Rv1460*stop mutant strains. Regions of homology that were cloned into the suicide delivery vector used for allelic exchange mutagenesis are denoted by hashed boxes and position of PCR screening primers scr1, 2 and 3 are indicated. (C) PCR screening results are 1 135 bp and 408 bp products for Δ*Rv1460*stop mutants and wild-type respectively. Southern blots using (D) NcoI digested chromosomal DNA and ‘*Rv1460* upstream’ region as probe and (E) ApaI digested chromosomal DNA and ‘*Rv1460* downdel’ region as probe.

**Figure D. Sanger sequencing of the deleted region in the *M. tuberculosis* truncation (Δ*Rv1460*stop) mutants.** Sanger sequencing of the region containing the deletion and the extra nucleotide causing a frameshift mutation in the Δ*Rv1460*stop_1.19, Δ*Rv1460*stop_5.19 and Δ*Rv1460*stop_5.20 truncation mutants. The black box indicates the new transcriptional start site of *Rv1460*, yellow and blue boxes indicate the position of the Rv1460upR and Rv1460stopF primers respectively (Table S4). The grey boxes indicate the extra nucleotide causing a frameshift mutation and the position of the premature stop codon it would cause.


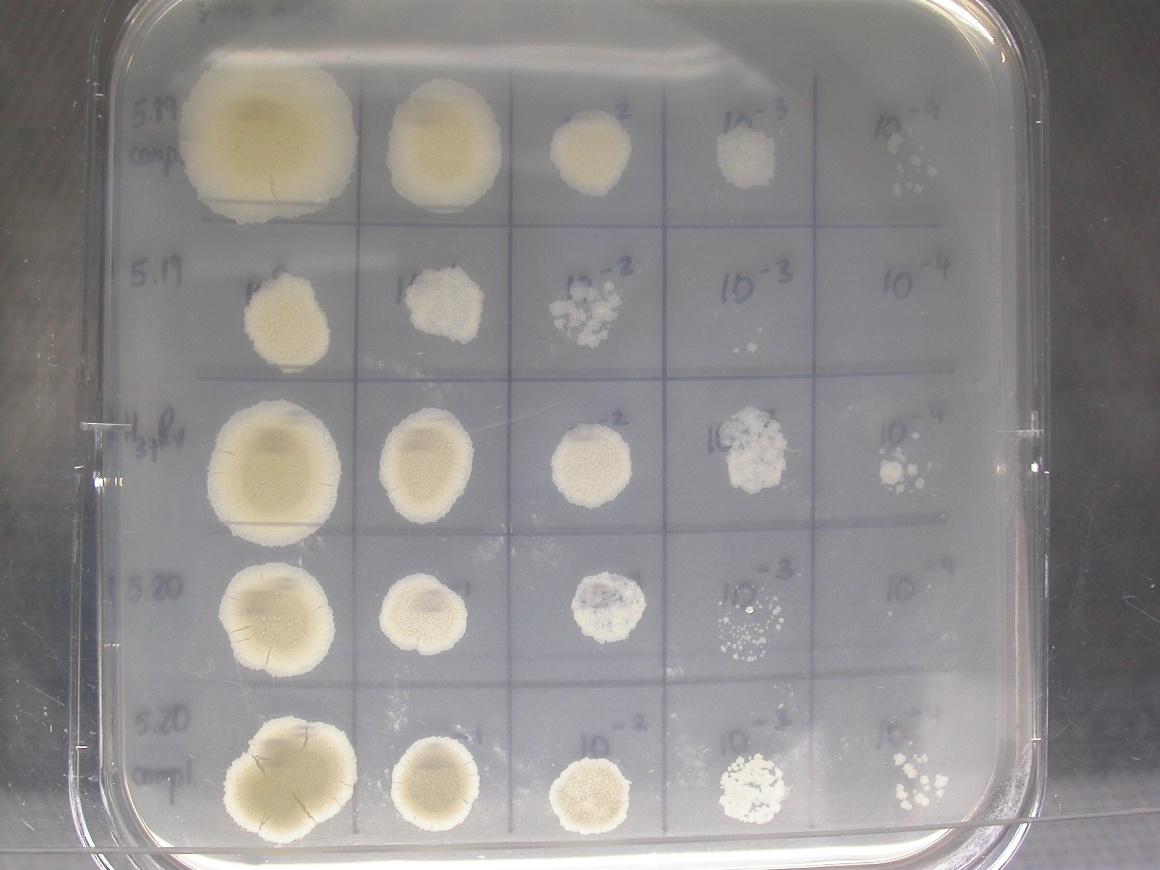


**Δ*Rv1460*stop_5.19 *attB*::pMVS1460**

**10^0^ 10^-1^ 10^-2^ 10^-3^ 10^-4^**

**Δ*Rv1460*stop_5.20 *attB*::pMVS1460**

**Δ*Rv1460*stop_5.20**

**Δ*Rv1460*stop_5.19**

**Δ*Rv1460*stop_5.20 *attB*::pMVS1460**

**Δ*Rv1460*stop_5.20**

**Δ*Rv1460*stop_5.19**

**Δ*Rv1460*stop_5.19 *attB*::pMVS1460**

**DAY 17**

**DAY 10**

**H37Rv**

**H37Rv**


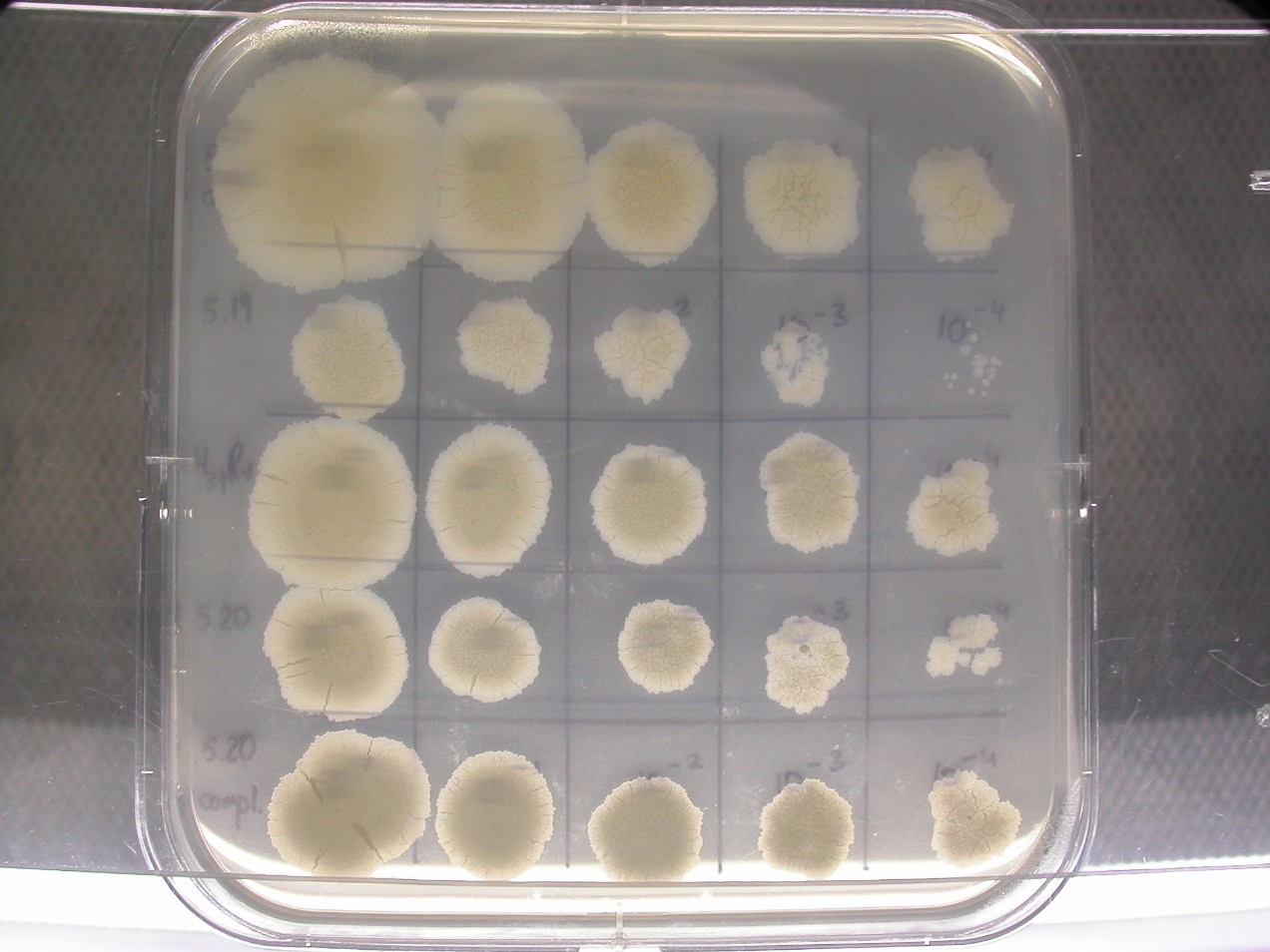


**Figure E. Truncation (Δ*Rv1460*stop) mutants are impaired for growth on solid media.** H37Rv (wild-type), Δ*Rv1460*stop_5.19, 5.20 and 1.19 mutants and their complemented strains were cultured in liquid media to an OD_600nm_ of approximately 0.2 and serial dilutions spotted onto solid media. Pictures show growth after 10 and 17 days. Continued overleaf.

**10^0^ 10^-1^ 10^-2^ 10^-3^ 10^-4^**


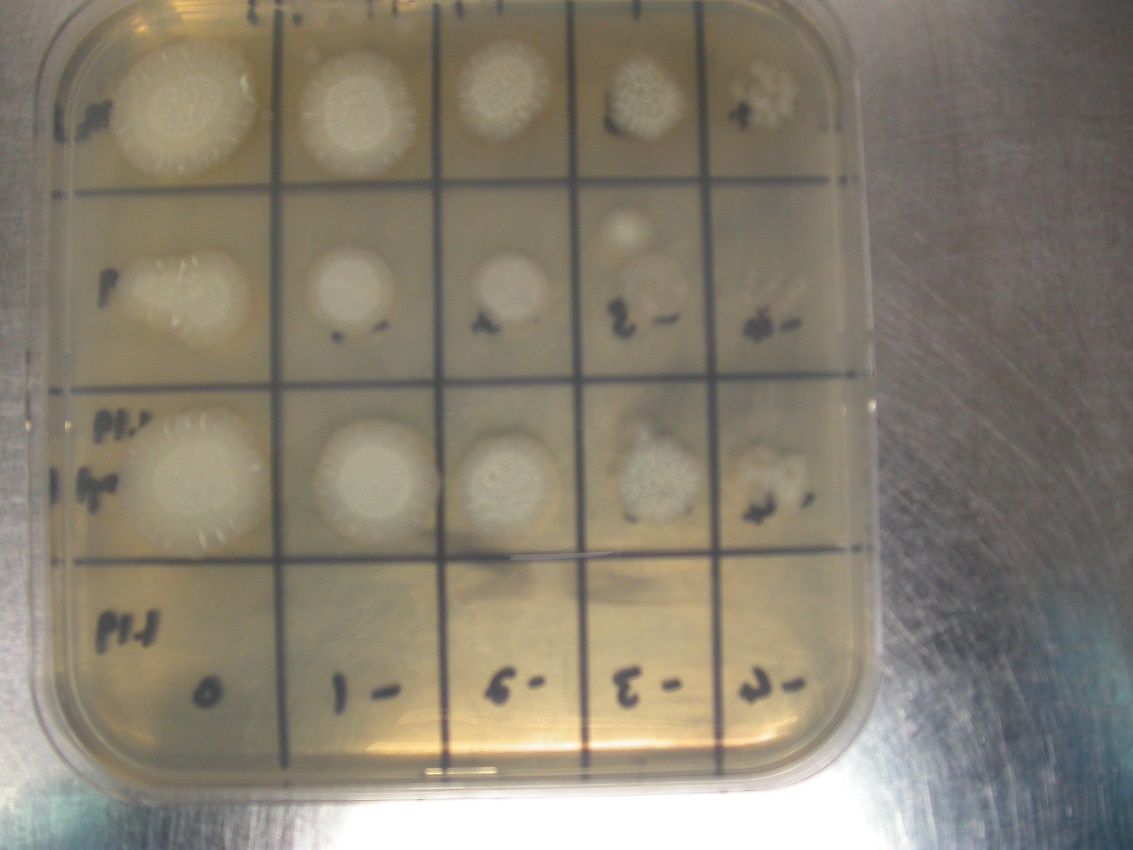

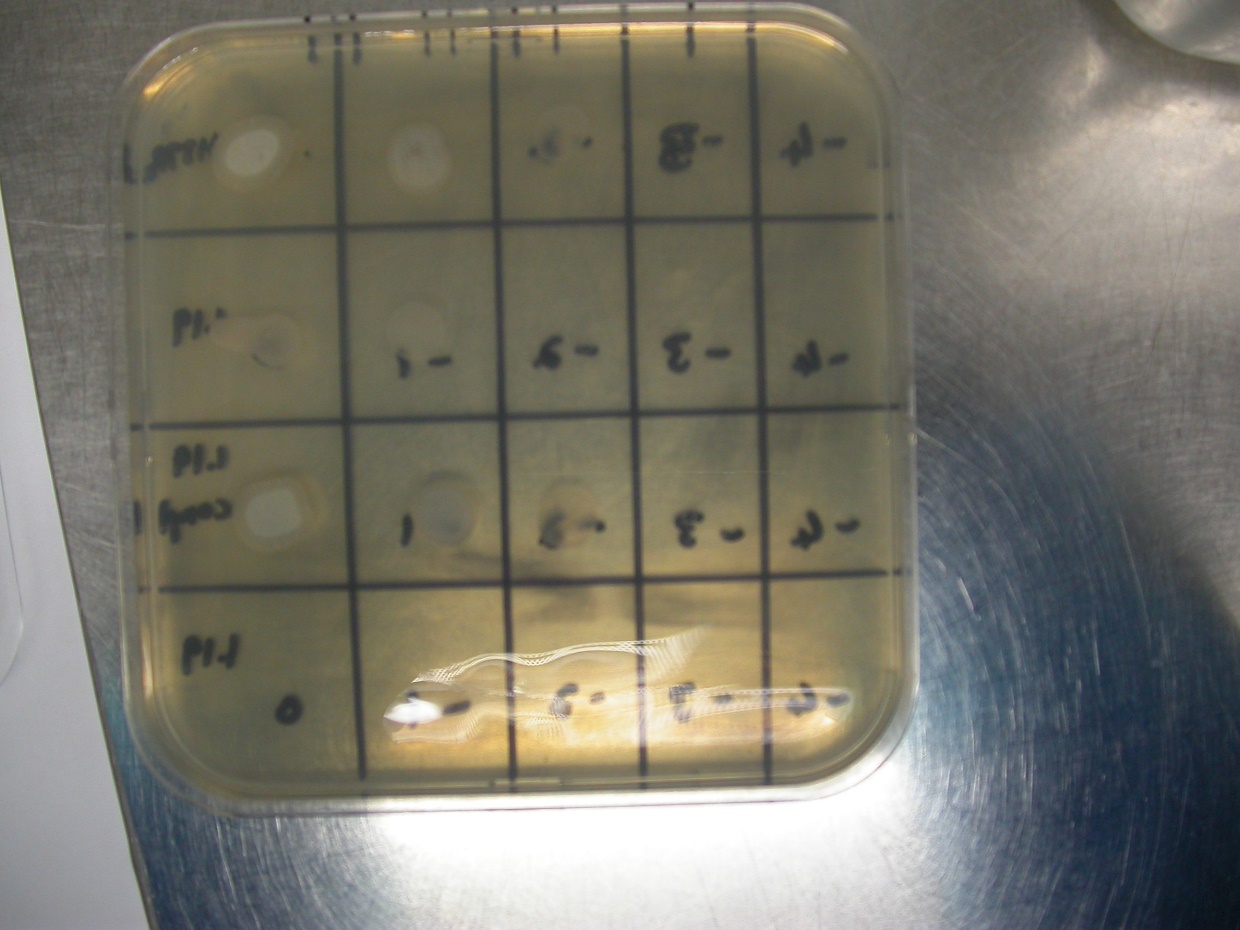


**Δ*Rv1460*stop_1.19 *attB*::pMVS1460**

**DAY 17**

**DAY 10**

**Δ*Rv1460*stop_1.19**

**Δ*Rv1460*stop_1.19**

**H37Rv**

**H37Rv**

**Δ*Rv1460*stop_1.19 *attB*::pMVS1460**

**Figure F. Truncation (Δ*Rv1460*stop) mutants are impaired for growth on solid media.** H37Rv (wild-type), Δ*Rv1460*stop_5.19, 5.20 and 1.19 mutants and their complemented strains were cultured in liquid media to an OD_600nm_ of approximately 0.2 and serial dilutions spotted onto solid media. Pictures show growth after 10 and 17 days.

**Figure G. PCR amplification of the (A) hygromycin (*hyg^r^*) and (B) kanamycin (*aph*) resistance genes.** Gels show examples of PCR screening for the (A) *hyg^r^* and (B) *aph* cassette of colonies recovered following transformation of Δ*Rv1460* *attB*::pMV1460 with either pCV-125 or pCV1460. Negative *hyg^r^* amplification and positive *aph* amplification indicates that pCV-125 and pCV1460 have replaced pMV1460 at the *attB* site in pCV-125 colonies 5 and 6 and pMV1460 colonies 1 and 2 respectively. Primers used for screening for *hyg^r^* are HYGF: ATCGGTGAAGCCGGAGAG and HYGR: AGGTCCGCTGTGACACAAGA (expected size 951 bp) and *aph* are aphF: CCACTGTTACAACCAATTAACCAAT and aphR: CATCATGAACAATAAAACTGTCTGCT (expected size 894 bp) respectively. pMV306H served as a positive control for *hyg^r^.*

**B**

**A**

**Figure H. Purification of recombinant Rv1460.** (A) Elution profile of recombinant Rv1460 from Sephadex S200 column. Position of calibration standards are indicated: γ-globulin (158 kDa), ovalbumin (44 kDa), myoglobin (17 kDa). (B) SDS-PAGE of gel filtration fractions. The elution times (in minutes) of the gel filtration fractions loaded are indicated above each lane. The expected size of N-terminally His-tagged Rv1460 monomer is indicated.


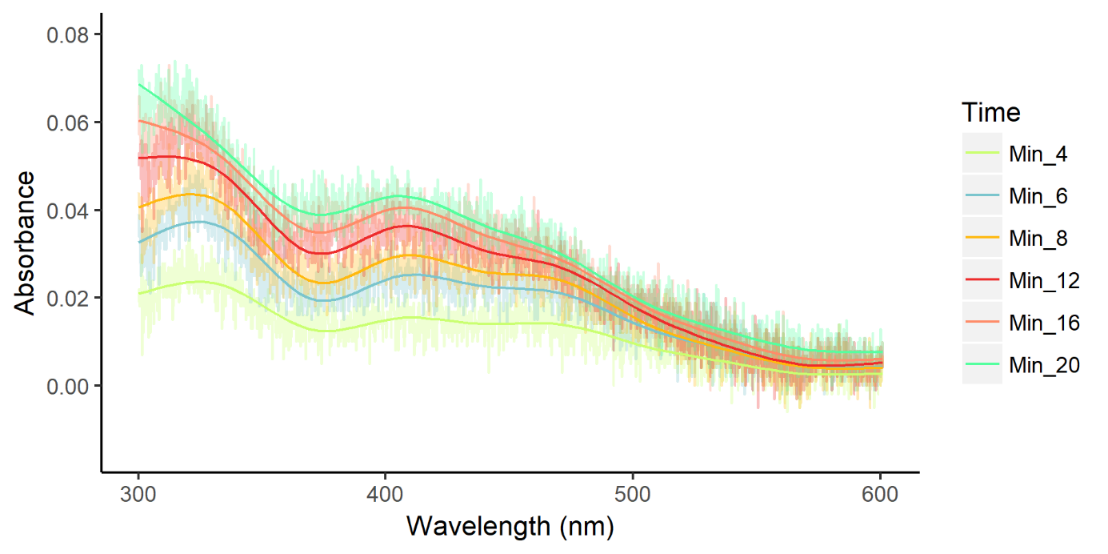

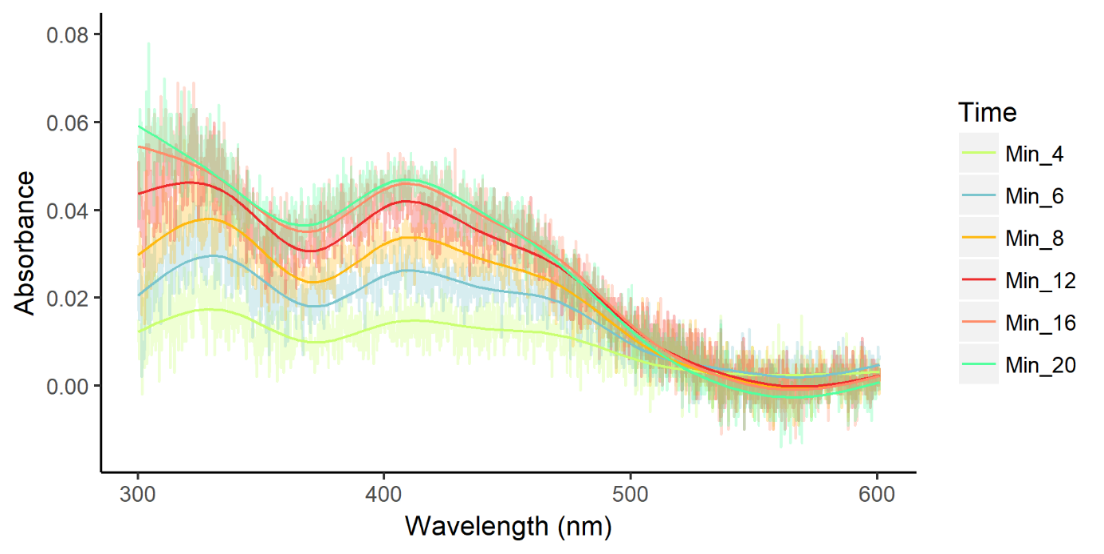


**C**

**B**

**A**

**Figure I. Enzymatic reconstitution using *E. coli* IscS and IscU.** (A) UV-visible spectrum of reconstitution reaction (A) containing Rv1460 (50 µM) or (B) without Rv1460 over 20 min. Both reactions contained (NH_4_)_2_Fe(SO_4_)_2_ (25 µM), DTT (3 mM), IscS*_E.coli_* (1 µM) and IscU*_E.coli_* (5 µM) in 20 mM Tris-HCl, 150 mM NaCl pH 8.0. The absorbance at 2 minutes was used as a blank for the reaction. Semi-transparent lines represent raw data points and solid line smoothers were added to emphasise the differences in distribution using statistical analysis language R version 3.4.1. (C) Near-UV CD spectra of the reactions containing Rv1460 (solid line) and without Rv1460 (dashed line).

**A**

**B**

**Figure J. Far-UV CD spectrum and thermal denaturation of apo- and holo-Rv1460.** (A) Far-UV CD spectrum for apo- and holo-Rv1460 at 0.116 mg/ml protein reconstituted using the lithium sulphide method followed by buffer exchange into gel filtration buffer. (B) Thermal denaturation monitored ellipticity at 222 nm between 5 and 91 ^o^C for apo- and holo-Rv1460.

**Figure K. UV-visible spectrum of chemical reconstitution of Rv1460 using lithium sulphide after purification by buffer exchange.** Rv1460 was reconstituted using the lithium sulphide method and reactions purified by buffer exchange. The reaction contained 23 µM Rv1460 (black) or no Rv1460 (grey).

**Table A. List of bacterial strains used and generated during this study**

| **Bacterial strains** | **Description** | **Source/reference** |
| --- | --- | --- |
| *Mycobacterium tuberculosis* |  |  |
| H37Rv | H37RvMA (ATCC: 27294) | [1] |
| *∆Rv1460 attB*::pMV1460 | Derivative of H37Rv carrying a 528bp unmarked in frame deletion in *Rv1460* generated in the presence of the pMV*Rv1460* vector integrated into the mycobacterial *attB* site; Hyg^r^ | This study |
| Rv1460 truncation mutants  Δ*Rv1460*stop_1.19  Δ*Rv1460*stop_5.19  Δ*Rv1460*stop_5.20 | Three independently isolated derivatives of H37Rv carrying a 236 bp unmarked deletion of the predicted *Rv1460* DNAbd followed by a frame-shift mutation causing a premature stop codon and truncation of the Rv1460 protein. These mutants were generated with the same allelic exchange substrate and harbour the same deletion. | This study |
| Δ*Rv1460*stop_1.19 *attB*::pMVS1460 | Derivatives of Δ*Rv1460*stop mutants carrying the pMV1460 vector stably integrated into the mycobacterial *attB* site | This study |
| Δ*Rv1460*stop_5.19 *attB*::pMVS1460 |  |  |
| Δ*Rv1460*stop_5.20 *attB*::pMVS1460 |  |  |
|  |  |  |
| *Mycobacterium smegmatis* |  |  |
| mc^2^ 155 | *ept-1* (efficient plasmid transformation) mutant of mc^2^6 | [2] |
| mc^2^ *attB::*pSE100 (pJEM15) | Derivative of mc^2^155 carrying pSE100 integrated at the *attB* locus and carrying a plasmid with a promoterless *lacZ* gene; Hyg^r^ Kan^r^ | This study |
| mc^2^ *attB::*pSE100 (pJEM100pro) | Derivative of mc^2^155 carrying pSE100 integrated at the *attB* locus and carrying a plasmid with a 139 bp promoter region upstream of the *lacZ* gene; Hyg^r^ Kan^r^ | This study |
| mc^2^ *attB::*pSE100 (pJEM300pro) | Derivative of mc^2^155 carrying pSE100 integrated at the *attB* locus and carrying a plasmid with a 314 bp promoter region upstream of the *lacZ* gene; Hyg^r^ Kan^r^ | This study |
| mc^2^ *attB::*pSE1460 (pJEM15) | Derivative of mc^2^155 with a plasmid expressing *Rv1460* integrated at the *attB* locus and carrying a plasmid with a 139 bp promoter region upstream of the *lacZ* gene; Hyg^r^ Kan^r^ | This study |
| mc^2^ *attB::*pSE1460 (pJEM100pro) | Derivative of mc^2^155 with a plasmid expressing *Rv1460* integrated at the *attB* locus and carrying a plasmid with a 139 bp promoter region upstream of the *lacZ* gene; Hyg^r^ Kan^r^ | This study |
| mc^2^ *attB::*pSE1460 (pJEM300pro) | Derivative of mc^2^155 with a plasmid expressing *Rv1460* integrated at the *attB* locus and carrying a plasmid with a 314 bp promoter region upstream of the *lacZ* gene; Hyg^r^ Kan^r^ | This study |

Continued on overleaf

**Table A. List of bacterial strains used and generated during this study (continued)**

| **Bacterial strains** | **Description** | **Source/reference** |
| --- | --- | --- |
| mc^2^ *attB::*pSE1460_C203S (pJEM300pro) | Derivative of mc^2^155 with a plasmid expressing *Rv1460* (carrying C203S mutation) integrated at the *attB* locus and carrying a plasmid with a 314 bp promoter region upstream of the *lacZ* gene; Hyg^r^ Kan^r^ | This study |
| mc^2^ *attB::*pSE1460_C216S (pJEM300pro) | Derivative of mc^2^155 with a plasmid expressing *Rv1460* (carrying C216S mutation) integrated at the *attB* locus and carrying a plasmid with a 314 bp promoter region upstream of the *lacZ* gene; Hyg^r^ Kan^r^ | This study |
| mc^2^ *attB::*pSE1460_C242S (pJEM300pro) | Derivative of mc^2^155 with a plasmid expressing *Rv1460* (carrying C242S mutation) integrated at the *attB* locus and carrying a plasmid with a 314 bp promoter region upstream of the *lacZ* gene; Hyg^r^ Kan^r^ | This study |
| mc^2^ *attB::*pSE1460_C244S (pJEM300pro) | Derivative of mc^2^155 with a plasmid expressing *Rv1460* ((carrying C244S mutation) integrated at the *attB* locus and carrying a plasmid with a 314 bp promoter region upstream of the *lacZ* gene; Hyg^r^ Kan^r^ | This study |
| mc^2^ *attB::* pSE1460_C203S/C216S/C244S (pJEM300pro) | Derivative of mc^2^155 with a plasmid expressing *Rv1460* (carrying C203S,C216S,C244S mutations) integrated at the *attB* locus and carrying a plasmid with a 314 bp promoter region upstream of the *lacZ* gene; Hyg^r^ Kan^r^ | This study |
|  |  |  |
| *Escherichia coli* |  |  |
| XL1 Blue | *recA1 endA1 gyrA96 thi-1 hsdR17 supE44 relA1 lac* [F ́ *proAB lacI*^q^Z∆*M15* Tn*10*; Tet^r^ | Stratagene |
| Arctic express (DE3) | B F^–^ *ompT hsdS*(r_B_^–^ m_B_^–^) *dcm*^+^ Tet^r^ *gal* λ(DE3) *endA* Hte [*cpn10 cpn60*]; Gent^r^ | Agilent Technologies |
| Rv1460 | Derivative of Arctic express (DE3) containing the pETM-11Rv1460nss plasmid for overexpression of Rv1460 as an N-terminally His-tagged protein | This study |
| BL21(DE3)pLysS | F^–^ *ompT hsdSB*(r_B_^–^ m_B_^–^) *gal dcm* (DE3) pLysS; Cam^r^) | Novagen |
| NifS | Derivative of BL21(DE3)pLysS containing the pDB845 vector for overexpression of  *A. vinelandii* NifS as a C-terminally 7×His-tagged protein | [3] |

**Table B. List of primers used for reverse transcription and amplification of *Rv1460*, *Rv1461* and *SigA* for qRT-PCR and ARF-TSS**

| **Gene** | **Primer name** | **Sequence (5’ → 3’)** | **Binding position^a^** | **Product size** |
| --- | --- | --- | --- | --- |
| *Rv1460* | Rv1460F^b^ | GAATTGTGCGAAACCGAGCAGCAG | 640 bp from start of *Rv1460* | 106 bp product |
|  | Rv1460R^b^ | CAGGGGTACGTGGGTGGTG | 732 bp from start of *Rv1460* |  |
|  | Rv1460RT | TCATCGGGACGCTCCTTCGG | 788 bp from start of *Rv1460* | NA |
| *Rv1461* | Rv1461F | CAAGAGCGTTGCCCAGCCC | 24 bp from start of *Rv1461* | 93 bp product |
|  | Rv1461R | GAGTCCGCCCAGCCGTAG | 99 bp from start of *Rv1461* |  |
|  | Rv1461RT | GCTCGTTCTTCTTCGCGGA | 175 bp from start of *Rv1461* | NA |
| *sigA* | SigAF | TGCAGTCGGTGCTGGACAC | 1379 bp from start of *sigA* | 195 bp product |
|  | SigAR | CGCGCAGGACCTGTGAGCGG | 1552 bp from start of *sigA* |  |
|  | SigART | CTGACATGGGGGCCCGCTACGTTG | 19 bp downstream of s*igA* | NA |
|  |  |  |  |  |
| *Rv1460* | 1460ARF-TSS | /5Phos/ATAGGAGTGGTCGAGCTTGG | 340 bp from start of *Rv1460* | NA |
|  | 1460ARF-F1 | CTGATCGAGGCGGGTGAC | 237 bp from start of *Rv1460* |  |
|  | 1460ARF-R1 | GGTGATCGATCCGGATTCCA | 154 bp from start of *Rv1460* | Variable |
| *Rv1461* | 1461ARF-TSS | /5Phos/GCTCGTTCTTCTTCGCGGA | 174 bp from start of *Rv1461* | NA |
|  | 1461ARF-F1 | CTTTCCGAGGCGGTGGTC | 147 bp from start of *Rv1461* |  |
|  | 1461ARF-R1 | GATCGCCTCTTCCTGGGTC | 59 bp from start of *Rv1461* | Variable |

^a^ Position indicated according to Tuberculist annotation of *Rv1460*

^b^ The gene specific *Rv1460* primers bind outside the region deleted in the *Rv1460stop* mutant and the transcript levels of *Rv1460* can thus still be assessed for the *Rv1460stop* mutant

**Table C. List of plasmids used and generated during this study**

| **Plasmids** | **Description** | **Source/ reference** |
| --- | --- | --- |
| p2NIL | Cloning vector; Kan^r^ | [4] |
| pGOAL17 | Plasmid with the *lacZ* and *sacB* genes as a PacI cassette; Amp^r^ | [4] |
| p2NIL17ΔRv1460 | Suicide delivery vector containing the upstream and downdel regions (Table S4) and PacI cassette from pGoal17; Kan^r^ | This study |
| p2NIL17*Rv1460*stop | Suicide delivery vector containing the upstream and downDNAbddelstop regions (Table S4) and PacI cassette from pGoal17, Kan^r^ | This study |
| pMV306H | Mycobacterial integrating shuttle vector pMV306 derivative containing a *hyg* gene; Hyg^r^ | [5] |
| pMV1460 | Derivative of pMV306H containing *Rv1460compl* (Table S4) | This study |
| pMVS1460 | Derivative of pMV1460 which has a 926 bp region containing the bulk of the integrase gene (103 bp at the 5’ end of the gene remaining) removed by restriction enzyme digestion with PstI and AvrII and religation of the blunted vector, creating stable integration into the mycobacterial *attB* site [6] | This study |
| pBluescript*int* | Used in combination with pMVS1460 to provide integrase for integration at the *attB* site. This vector does not have a mycobacterial origin of replication and is therefore lost upon replication thereby removing the integrase and ensuring stable integration of the pMVS1460 vector when it is co-transformed | [7] |
| pJET1.2 | Linearised blunt end cloning vector; Amp^r^ | CloneJet |
| pJET*Rv1460*N | Derivative of pJET1.2 containing the *Rv1460*N region (Table S4) cloned into the blunt end cloning position | This study |
| pET28a | *E. coli* expression plasmid with a T7 *lac* promoter and a multiple cloning site for creating N- or C- terminally His-tag fusion protein with a thrombin cleavage site for the removal of the N-terminal His-tag; Kan^r^ | Novagen |
| pET28*Rv1460*N | Derivative of pET28a with the *Rv1460*N fragment (Table S4) cloned into the multiple cloning site between NdeI and HindIII cut sites | This study |
| pET28*Rv1460*C203S  pET28*Rv1460*C216S  pET28*Rv1460*C242S  pET28*Rv1460*C244S  pET28*Rv1460*C203S/  C216S/C244S | Series of expression vectors derived from pET28*Rv1460*N harbouring C203S, C216S, C242S, C244S substitutions in Rv1460. These vectors were used for the generation of pSE1460_C203S, C216S, C242S and C244S vectors. | This study |

Continued on overleaf

**Table C. List of plasmids used and generated during this study (continued)**

| **Plasmids** | **Description** | **Source/ reference** |
| --- | --- | --- |
| pETM-11 | *E. coli* expression plasmid with a T7 *lac* promoter and a multiple cloning site for creating N- or C- terminally His-tag fusion protein with a TEV cleavage site for the removal of the N-terminal His-tag; Kan^r^ | Lab stock |
| pETM-11Rv1460nss | Derivative of pETM-11 with the *Rv1460nss* fragment (Table S4) cloned into the multiple cloning site between NcoI and HindIII cut sites | This study |
| pDB845 | *E. coli* expression plasmid with a T7 *lac* promoter for expression of *A. vinelandii* NifS as a C-terminally 7×His-tagged protein | [3] |
|  |  |  |
| pSE100 | Vector for the expression of genes from a PmycTetO promoter, high copy number in *E. coli* and low in mycobacteria; Hyg^r^ (Addgene: Plasmid #17972) | [8] |
| pSE1460 | Derivative of pSE100 with *Rv1460RBS* cloned between BamHI and HindIII and expressed from the PmycTetO promoter | This study |
| pSE1460_C203S  pSE1460_C216S  pSE1460_C242S  pSE1460_C244S  pSE1460_C203S/C216S/  C244S | Series of mycobacterial expression vectors derived from pSE1460 harbouring C203S, C216S, C242S, C244S substitutions in Rv1460 | This study |
| pCV-125 | Mycobacterial integrating shuttle vector containing a promoterless *lacZ* gene; Kan^r^ | [9] |
| pCV1460 | Derivative of pCV-125 from which the *lacZ* gene was removed and *Rv1460* under the expression of the PmycTetO promoter cloned between EcoRV and BstZ171 sites. | This study |
| pCV1460_C203S  pCV1460_C216S  pCV1460_C242S  pCV1460_C244S  pCV1460_C203S/C216S/C244S | Series of mycobacterial expression vectors derived from pCV1460 harbouring C203S, C216S, C242S, C244S substitutions in Rv1460. These vectors were generated by sub-cloning the promoter and gene (BcuI/SalI digest) from each pSE1460 vector into the pCV-125 backbone (EcoRV/Bst171 digest) | This study |
| pJEM15 | Mycobacterial episomal shuttle vector containining a promoterless *lacZ* gene; Kan^r^ | [10] |
| pJEM139pro | Derivative of pJEM15 with the 139 bp promoter region upstream of *Rv1460* cloned into the BamHI site | This study |
| pJEM314pro | Derivative of pJEM15 with 314 bp, containing promoter region (139 bp) and first 175 bp of *Rv1460*, cloned into the BamHI site | This study |

**Table D. List of primers used for suicide delivery vector generation, complementation and Rv1460 expression in *E. coli***

| **Region** | **Primer name** | **Sequence (5’ → 3’)^a^** | **Description** | **Mutation^b^** | |
| --- | --- | --- | --- | --- | --- |
| *Rv1460* upstream | Rv1460upF | GGATCCGGGTGAGTGACAACACG (BamHI) | 1482 bp product, including a 1365 bp region upstream of *Rv1460* and 105 bp of the 5’ end of *Rv1460* |  | |
|  | Rv1460upR | GGTACCGGGGACAGTGGTAGAGACC (Asp718) |  |  |  |
| *Rv1460* downdel | Rv1460delF | ggtaccttccccgaattgtgcg (Asp718) | 1106 bp product, including 174 bp of the 3’ end of *Rv1460* and 919 bp downstream of *Rv1460* | Used in combination with the *Rv1460* upstream region to delete 175 of the 268 amino acids of the Rv1460 protein (codons 36 to 211 deleted). | |
|  | Rv1460delR | GAAGCTTGACATCGGTGTGAAGGTGAA (HindIII) |  |  |  |
|  |  |  |  |  |  |
| *Rv1460* downDNAbddelstop | Rv1460stopF^c^ | GGTACC**C**AAGCTCGACCACTCCTAT (Asp718) | 1398 bp product including 466 bp of the 3’ end of *Rv1460* and 919 bp downstream of *Rv1460* | Used in combination with Rv1460 upstream region to delete part of the predicted DNA-binding domain of Rv1460 (codons 36 to 114 deleted) and introduce a premature stop codon at codon 122. | |
|  | Rv1460delR | GAAGCTTGACATCGGTGTGAAGGTGAA (HindIII) |  |  |  |
| *Rv1460compl* | ComplF | GGATATCAGCTCGGTAGTGGTCAGCG (EcoRv) | 975 bp product including  139 bp upstream of *Rv1460*, presumably including the promoter region, up to 15 bp downstream of *Rv1460* | NA | |
|  | ComplR | GAAGCTTCCTCTGGGGTGAGTGTCATC (HindIII) |  |  |  |
|  |  |  |  |  |  |
| Rv1460RBS^d^ | TetnativeF | GGATCCTGCTTA**GGCGA**TCTCAATGTG (BamHI) | 18 bp upstream up to 16 bp downstream of Rv1460 including a possible native ribosomal binding site | NA |  |
|  | ComplR | GAAGCTTCCTCTGGGGTGAGTGTCATC (HindIII) |  |  |  |

Continued on overleaf

**Table D. List of primers used for suicide delivery vector generation, complementation and Rv1460 expression in *E. coli* (continued)**

| **Region** | **Primer name** | **Sequence (5’ → 3’)^a^** | **Description** | **Mutation^b^** |  |
| --- | --- | --- | --- | --- | --- |
| Suicide delivery vectors | SeqF0 | TACCGGCATAACCAAGCCTA | Sequencing final suicide delivery vectors | NA | |
| Suicide delivery vectors | SeqF1 | TGCCTGACTGCGTTAGCAAT | Sequencing final suicide delivery vectors | NA |  |
| Suicide delivery vectors | SeqF2 | TGTCCTCTGGCAAATCATCC | Sequencing final suicide delivery vectors | NA |  |
| Suicide delivery vectors | SeqF3 | TGATCGGTCTACCAGGGATG | Sequencing final suicide delivery vectors | NA |  |
| Suicide delivery vectors | SeqF3b | ATTTGCACACCGTGAATCG | Sequencing final suicide delivery vectors | NA |  |
| Suicide delivery vectors | SeqF4 | CATATGGTCCGCTGTTCTTGT | Sequencing final suicide delivery vectors | NA |  |
|  | | | | |  |
| *Rv1460*N^e^ | Rv1460pETNF | CGCATATG**GTG**ACCAGCACAACCCTGCCG (NdeI) | 821 bp product including the *Rv1460* gene according to the tuberculist annotation (including the stop codon) | NA |  |
|  | Rv1460pETNR | CAAGCTT**CAT**CGGGACGCTCCTTCGGTGCTG (HindIII) |  |  |  |
|  |  |  |  |  |  |
| C203S^f^ | SNP1F | CAGCACCAT**TCC**CCGGTATCC | Site directed mutagenesis | C→S at codon 203 in Rv1460 |  |
|  | SNP1R | GGATACCGG**GGA**ATGGTGCTG |  |  |  |
| C216S^f^ | SNP2F | CCCGAATTG**TCC**GAAACCGAG | Site directed mutagenesis | C→S at codon 216 in Rv1460 |  |
|  | SNP2R | CTCGGTTTC**GGA**CAATTCGGG |  |  |  |
| C242S^f^ | SNP3F | AACGGAGAC**TCC**GCCTGCACC | Site directed mutagenesis | C→S at codon 242 in Rv1460 |  |
|  | SNP3R | GGTGCAGGC**GGA**GTCTCCGTT |  |  |  |
| C244S^f^ | SNP4F | GACTGCGCC**TCC**ACCACCCAC | Site directed mutagenesis | C→S at codon 244 in Rv1460 |  |
|  | SNP4R | GTGGGTGGT**GGA**GGCGCAGTC |  |  |  |

Continued on overleaf

**Table D. List of primers used for suicide delivery vector generation, complementation and Rv1460 expression in *E. coli* (continued)**

| **Region** | **Primer name** | **Sequence (5’ → 3’)^a^** | **Description** | **Mutation^b^** |
| --- | --- | --- | --- | --- |
| Rv1460nss | Rv1460nssFor | CCA TG **GTG** AAA ATC CCG GCG GTC TC (NcoI) | 746 bp region including the *Rv1460* gene according to the new transcriptional start site at position +73 (including the stop codon) | NA |
|  | Rv1460pETNR | CAAGCTT**CAT**CGGGACGCTCCTTCGGTGCTG (HindIII) |  |  |
|  |  |  |  |  |
| EMSA314Rv1460 | EMSA314Rv1460F | GCTCTAGAAGCTCGGTAGTGGTCAGCG | 314 bp product including 139 bp upstream of *Rv1460* and the first 175 bp of *Rv1460* | NA |
|  | EMSA314Rv1460R | GCGGATCCGGTGATCGATCCGGATTCCA |  |  |
| EMSA139Rv1460 | EMSA139Rv1460F | **GCTCTAGAAGCTCGGTAGTGGTCAGCG** | 139 bp region upstream of *Rv1460* | NA |
|  | EMSA139Rv1460R | **GTCGGATCCATTGAGATCGCCTAAGCAATTC** |  |  |
| EMSA139Rv1460 | EMSA139Rv1460F | GCTCTAGAAGCTCGGTAGTGGTCAGCG | 139 bp region upstream of *Rv1460* | NA |
|  | EMSA139Rv1460R | GTCGGATCCATTGAGATCGCCTAAGCAATTC |  |  |

^a^ RE cutting sites are underlined and RE indicated in parenthesis

^b^ For an in depth description of the *Rv1460* mutants generated refer to Fig S2

^c^ Extra nucleotide causing frame shift mutation indicated in bold

^d^ Native ribosomal binding site indicated in bold

^e^ Bold region indicating start and stop codons

^f^ Double underlined letter indicates the base pair change G→C introduced and bold letters indicate the codon which is mutated from C→S

**Table E. List of primers used for screening of SCO orientation and genotyping of *M. tuberculosis* *Rv1460* mutant strains**

| **Region** | **Primer name** | **Sequence (5’ → 3’)** | **Description** | **Expected product sizes** | | | **SCO orientation** | |
| --- | --- | --- | --- | --- | --- | --- | --- | --- |
|  |  |  |  | **WT** | ***ΔRv1460*** | ***Rv1460stop*** | **Upstream SCO** | **Downstream SCO** |
| SCO region 1 | SCOupup | CAATAACAGCCAGCACACCA | Binds 49 bp upstream of upstream region and within downdel region | 2142 bp | 1624 bp | 1916 bp | 1624 bp | 2142 bp |
|  | SCOindel | GCAGTCTCCGTTGACGATG |  |  |  |  |  |  |
| SCO region 2 | SCOinup | CATCCCTGGTAGACCGATCA | Binds 76 bp of the 3’ end of the upstream region up to 105 bp downstream of downdel region | 1803 bp | 1287 bp | 1579 bp | 1803 bp | 1287 bp |
|  | SCOdowndel | ACTTCAGCCTTCCACCCATT |  |  |  |  |  |  |
|  |  |  |  |  |  |  |  |  |
| DCO genotyping | Scr1 | GATTCGTGACGGCAGATTGAGC | Binds within the upstream region | 408 bp | 843 bp | 1135 bp | NA | NA |
|  | Scr2 | GAGTGTGACCGTCCGAGACTG | Binds within the DNAbd region deleted in all mutants |  |  |  | NA | NA |
|  | Scr3 | CGATGCCATCGAGGTTGGAGC | Binds within the undeleted 174 bp region at 3’ end of *Rv1460* |  |  |  | NA | NA |

**Table F. Intracellular iron levels after three growth cycles in MM or MM + Fe^+3^**

|  |  | **µmol intracellular iron/g total protein** | | | |
| --- | --- | --- | --- | --- | --- |
| **Strain** | **Media** | **Replicate 1** | **Replicate 2** | **Replicate 3** | **Average** |
| H37Rv | MM + Fe^+3^ | 71.68 | 111.98 | 71.57 | 85.08 |
|  | MM | 23.65 | 30.59 | 10.04 | 21.43 |
| Δ*Rv1460*stop_1.19 | MM + Fe^+3^ | 59.72 | 152.55 | 61.25 | 91.17 |
|  | MM | 26.37 | 79.03 | 17.96 | 41.12 |
| Δ*Rv1460*stop_1.19 *attB*::pMVS1460 | MM + Fe^+3^ | 50.55 | 94.12 | 89.09 | 77.92 |
|  | MM | 33.15 | 36.80 | 5.30 | 25.08 |
| Δ*Rv1460*stop_5.19 | MM + Fe^+3^ | 56.62 | 135.42 | 51.86 | 81.30 |
|  | MM | 22.74 | 41.06 | 7.81 | 23.87 |
| Δ*Rv1460*stop_5.19 *attB*::pMVS1460 | MM + Fe^+3^ | 48.33 | 89.61 | 110.03 | 82.66 |
|  | MM | 36.13 | 26.48 | 2.61 | 21.74 |
| Δ*Rv1460*stop_5.20 | MM + Fe^+3^ | 44.51 | 87.11 | 62.15 | 64.59 |
|  | MM | 23.75 | 19.45 | 14.66 | 19.29 |
| Δ*Rv1460*stop_5.20 *attB*::pMVS1460 | MM + Fe^+3^ | 57.16 | 120.39 | 145.47 | 107.67 |
|  | MM | 31.84 | 28.02 | 4.81 | 21.56 |

**Supporting information references**

- - - 1. Ioerger TR, Feng Y, Ganesula K, Chen X, Dobos KM, Fortune S, et al. Variation among genome sequences of H37Rv strains of *Mycobacterium tuberculosis* from multiple laboratories. J Bacteriol 2010;192:3645–53.
      2. Snapper SB, Melton RE, Mustafa S, Kieser T, Jacobs WR Jr. Isolation and characterization of efficient plasmid transformation mutants of *Mycobacterium smegmatis*. Mol Microbiol 1990;4:1911–9.
      3. Zheng L, White RH, Cash VL, Jack RF, Dean DR. Cysteine desulfurase activity indicates a role for NIFS in metallocluster biosynthesis. Proc Natl Acad Sci U S A. 1993 Apr 1;90(7):2754–8.
      4. Parish T, Stoker NG. Use of a flexible cassette method to generate a double unmarked *Mycobacterium tuberculosis* *tlyA* *plcABC* mutant by gene replacement. Microbiology 2000;146:1969–75.
      5. Stover CK, de la Cruz VF, Fuerst TR, Burlein JE, Benson LA, Bennett LT, et al. New use of BCG for recombinant vaccines. Nature 1991;351:456–60.
      6. Edelheit O, Hanukoglu A, Hanukoglu I. Simple and efficient site-directed mutagenesis using two single-primer reactions in parallel to generate mutants for protein structure-function studies. BMC Biotechnol 2009;9:61–8.
      7. Springer B, Sander P, Sedlacek L, Ellrott K, Böttger EC. Instability and site-specific excision of integration-proficient mycobacteriophage L5 plasmids: development of stably maintained integrative vectors. Int J Med Microbiol 2001;290:669–75.
      8. Guo XV, Monteleone M, Klotzsche M, Kamionka A, Hillen W, Braunstein M, et al. Silencing *Mycobacterium smegmatis* by using tetracycline repressors. J Bacteriol 2007;189:4614–23.
      9. Alland D, Steyn AJ, Weisbrod T, Aldrich K, Jacobs WR. Characterization of the *Mycobacterium tuberculosis* *iniBAC* promoter, a promoter that responds to cell wall biosynthesis inhibition. J Bacteriol 2000;182:1802–11.
      10. Timm J, Lim EM, Gicquel B. *Escherichia coli*-mycobacteria shuttle vectors for operon and gene fusions to *lacZ*: the pJEM series. J Bacteriol 1994;176:6749–53.
